# Computational Fluid Dynamics of the Flow of the Deformable Toroidal Embolic Agents Within Straight and Stenotic Pipes by Full Eulerian FSI Method

**DOI:** 10.1002/cnm.70089

**Published:** 2025-09-08

**Authors:** Kazuki Matsumiya, Kazuyasu Sugiyama, Natsuko F. Inagaki, Shu Takagi, Taichi Ito

**Affiliations:** ^1^ Department of Chemical System Engineering School of Engineering, The University of Tokyo Tokyo Japan; ^2^ Department of Mechanical Science and Bioengineering, Graduate School of Engineering Science The University of Osaka Osaka Japan; ^3^ Department of Bioengineering School of Engineering, The University of Tokyo Tokyo Japan; ^4^ Department of Mechanical Engineering School of Engineering, The University of Tokyo Tokyo Japan; ^5^ Department of Radiology and Biomedical Engineering School of Medicine, The University of Tokyo Tokyo Japan

**Keywords:** deformation analysis, fluid–structure interaction, shape anisotropy, torus, transcatheter arterial embolization, volume of fluid

## Abstract

The effect of shape and size of embolic agents on embolization phenomena has been discussed clinically for transcatheter arterial chemoembolization (TACE). We numerically discussed the unique embolization behavior of new deformable toroidal microparticles in blood vessels by computational fluid dynamics simulations. We employed an Eulerian–Eulerian (full Eulerian) fluid–structure interaction (FSI) method to analyze the flow and deformation behaviors of a deformable torus in a cylindrical pipe. This method, based on the volume of fluid (VOF) method, is implemented in OpenFOAM and is verified by deformation tests with a visco‐hyperelastic material in cavity flow. The torus exhibits multiple steady states depending on initial orientation, position, shear modulus, and the aspect ratio between major and minor radii, and the rotation angles of inclined tori reach approximately 80°. Deformation analysis of cross‐sections reveals multiple deformation modes such as bending, rotation, and elongation over time. The equilibrium position of the torus is determined by the balance of various lift forces and becomes complex due to increased rotational diameter from elongation. Additionally, vortex structures and pressure gradients elucidate the mechanism that inclined tori are faster than horizontally oriented tori due to their deformation. Finally, flow tests of different microparticle shapes with the same surface area in a stenotic pipe show that the torus has the lowest pressure drop and flow rate reduction. These quantitative predictions are suggestive and encourage experimental study of toroidal microparticles as novel embolic agents in the future.

## Graphical abstract

We numerically analyzed the flow and deformation of a toroidal embolic agent in straight and stenotic pipes with full Eulerian fluid‐structure interaction. The fluid passes through the hole of the torus, and the deformation is categorized into three deformation modes such as bending, rotation, and elongation. Initially inclined torus shows the less pressure gradient compared with the horizontally and vertically centered tori due to their deformation over time.

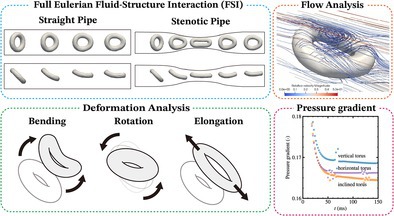

**Abbreviations**
CFD: computational fluid dynamics
CFL: Courant–Friedrichs–Lewy
DIC: diagonal‐based incomplete Cholesky
FSI: fluid–structure interaction
PCG: preconditioned conjugate gradient
PISO: pressure implicit split operator
PLIC: piecewise linear interface construction
PVA: polyvinyl alcohol
RDF: reconstruction distance function
SIMPLE: semi‐implicit methods for pressure linked equations
TACE: transcatheter arterial chemoembolization
TAE: transcatheter arterial embolization
TARE: transcatheter arterial radioembolization
UAE: uterine artery embolization
VOF: volume of fluid
WENO: weighted essentially nonoscillatory

## 1 Introduction

Transcatheter arterial embolization (TAE) represents a minimally invasive treatment to dose embolic and contrast agents to targeted arteries via catheterization, thereby inducing ischemia through the process of embolization [1]. TAE is applicable across a diverse range of medical conditions, including acute hemorrhage, aneurysms, arteriovenous malformations, and both benign and malignant tumors. In addition, transcatheter arterial chemoembolization (TACE) which employs drug‐loaded embolic agents, and transcatheter arterial radioembolization (TARE) utilizing radioactive embolic agents, are also effective approaches for the treatment of hepatocellular carcinoma [2, 3]. Although TAE and its derivative methods are effective in inducing ischemia for the treatment of hepatocellular carcinoma, they are associated with complications such as gastrointestinal bleeding due to portal hypertension and acute liver or renal failure resulting from thromboembolism [4, 5].

The effect of shape and size of embolic agents on embolization phenomena has been discussed clinically. Gelatin sponges and spherical hydrogel microparticles composed of poly(vinyl alcohol) (PVA) are widely used in clinics, and several in vivo and clinical studies have investigated the effect of embolization on the shape and deformability of these embolic agents [6]. In a comparative study on humans by Chua et al., uterine artery embolization (UAE) was performed on 17 patients undergoing myomectomy, revealing that trisacryl gelatin microspheres penetrated deeper into leiomyomata compared to nonspherical PVA microparticles [7]. Additionally, a double‐blinded randomized controlled trial by Yu et al. involving 56 patients undergoing UAE demonstrated that highly deformable PVA microspheres occluded more distal positions compared to trisacryl gelatin microspheres [8]. However, the high compressibility of PVA microspheres resulted in lower stability of embolization in urinary arteries over the long term. These clinical studies, which utilized commercially available embolic microparticles, did not closely examine the effects of viscoelasticity and shape anisotropy of the materials. Investigating these properties is expected to enhance therapeutic efficacy and expand the selection of embolic agents, thereby increasing the applicability of embolization therapy to a wider range of clinical cases.

Recently, shape anisotropy in microparticles is gaining significant attention in biomaterials research such as drug delivery and tissue engineering due to anisotropic characteristics such as enhanced deformability and increased specific surface area compared to spherical microparticles [9]. Luo et al. fabricated shape‐anisotropic PVA microparticles using microfluidic synthesis for TAE [10]. These capsule‐shaped PVA microparticles occluded rabbit arteries at more distal points compared to spherical ones. In a numerical study, Talebibarmi et al. simulated embolism by shape‐anisotropic blood emboli in cerebral arteries, corroborating Luo's findings [11]. On the other hand, we prepared drug‐loaded toroidal alginate hydrogel microparticles by electrospray method and analyzed the drug eluting mechanism [12]. Toroidal shape is expected to achieve partial embolization by the flow of the center hole of the particles, bringing to control the severity of hypoxia. These toroidal microparticles have potential for better‐controlled TACE in the future.

Numerical simulations are as effective as experimental approaches, which can evaluate the flow and deformation behavior of deformable toroidal microparticles at the low Reynolds numbers characteristic of arteries and arterioles. For analyzing the deformation of soft materials in fluid systems, numerical studies of fluid–structure interaction (FSI) are extensively employed, with various approaches generally categorized into partitioned and monolithic methods [13]. Notably, numerous numerical studies aim to elucidate the deformation of red blood cells and the rheological properties of blood using FSI methods [14]. Wang et al. systematically classified the equilibrium states of red blood cells across a wide range of shear rates using the immersed boundary method [15]. They highlighted that the equilibrium state depends not only on the flow rate but also on parameters such as the initial position, tube diameter, shear modulus, and internal viscosity. Additionally, Cetin and Sahin analyzed the deformation of red blood cells with finite membrane thickness using a monolithic solver based on the arbitrary Lagrangian–Eulerian formulation [16]. They reported that parachute‐like cells in small capillaries exhibited cupcake‐shaped buckling instability and that cell deformation was not axisymmetric but three‐dimensional. Consequently, deformable anisotropic microparticles in low Reynolds number flows, such as those in small blood vessels, have the potential to exhibit multiple stable equilibrium states and three‐dimensional deformation. However, to the best of our knowledge, no previous numerical studies have reported on the deformation of toroidal microparticles in fluid.

The aim of this study is to analyze the flow and deformation behavior of toroidal microparticles (hereinafter referred to as torus or tori) using monolithic FSI methods and to evaluate their applicability to blood vessel embolization. We employed a FSI method based on a Eulerian–Eulerian formulation (full Eulerian FSI) as reported by Sugiyama et al. [17]. and implemented the FSI solver in OpenFOAM, a C++‐based open‐source computational fluid dynamics (CFD) toolbox [18]. The full Eulerian FSI method is based on the Volume of Fluid (VOF) method [19] and utilizes fixed unstructured grids, which reduces the computational costs for remeshing and potentially prevents divergence due to large deformations. The numerical results indicate that the equilibrium state of the torus is mainly influenced by its initial orientation and the inclined torus anisotropically deforms in the pipe due to the complexity of some lift forces and vortex structures. The elongation and bending behaviors of the torus also depend on its geometric and physical properties, such as the shear modulus and the aspect ratio between the major and minor radii of the torus. Finally, we evaluated the embolization process of different shapes of microparticles with the same surface area within a stenotic pipe as a model of blood vessel.

## 2 Methodology

### 2.1 Geometry Model

**FIGURE 1:**
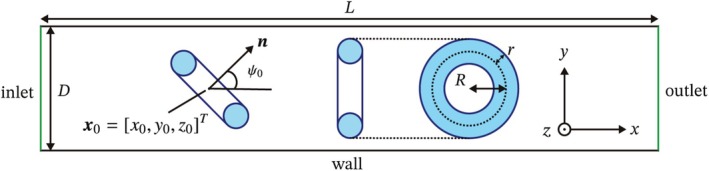
Schematic illustration of the cross‐section of the geometry in this simulation. The cylindrical pipe has a diameter D and length L and is composed of three boundaries: inlet, outlet, and wall. A torus has a small radius r and a large radius R, and is centered at the initial position $x_{0}$ and the initial orientation $\psi_{0}$. The orientation of a torus is defined by the angle between the orientation vector n and x‐axis.

First, the arteriovenous structure used in TAE is complex and varies among patients [20]. However, in this study, a symmetric cylinder is employed to evaluate the fundamental characteristics of torus flow and deformation. Figure 1 illustrates a cross‐section of the simulation area. The cylindrical pipe has a length (*L*) and a diameter (*D*). The torus shape is defined by its major radius (*R*) and minor radius (*r*). Additionally, the aspect ratio between major and minor radii of the torus (*R/r*) serves as an important nondimensional parameter for characterizing torus properties [21]. The orientation vector (n) is defined as a unit vector oriented along the axis of revolution of the torus. The initial condition of a torus is specified by the position of the centroid ($x_{0}={\left[x_{0},y_{0},z_{0}\right]}^{T}$) and its orientation described by the angle ($\psi_{0}$) between the *x*‐axis and orientation vector of the torus. The sides of the pipe are referred to as the wall, while the ends of the pipe are referred to as the inlet and outlet, respectively.

### 2.2 Governing Equations

The present study investigates the flow of an isothermal, incompressible, Newtonian fluid and an incompressible, visco‐hyperelastic solid which has internal viscosity and elasticity. It deforms without changing its volume and moves through the fluid with satisfying mass and momentum conservation. The full Eulerian FSI method incorporating the VOF method was detailed in a previous study [17]. The governing equations consist of mass and momentum conservation:

(1) ∇·u=0

(2) $$\begin{array}{c} \rho \left\{\partial_{t}u+\left(u\cdot \nabla \right)u\right\}=\nabla \cdot \sigma +\rho g \end{array}$$

where ***u*** is a velocity vector, *t* is a time, ρ is a density, σ is a Cauchy stress tensor, and ***g*** is a gravitational acceleration vector. The densities of fluid and solid ($\rho_{f},\rho_{s}$) are assumed to be equal as the density of alginate hydrogel closely matches that of plasma. In the VOF method, the multiphase fluid viscosity is described as the volume fraction‐weighted average of the solid (α) as follows:

(3) $$\begin{array}{c} \mu =\alpha \mu_{s}+\left(1-\alpha \right)\mu_{f} \end{array}$$

(4) $$\begin{array}{c} \alpha =\frac{1}{V_{i}}\int_{V_{i}}I\left(x,t\right)\mathrm{dV}=\left\{\begin{array}{c} 1,\\ 0<\alpha <1,\\ 0, \end{array}\right.\;\begin{array}{c} \text{solid}\\ \text{interface}\\ \text{fluid} \end{array} \end{array}$$

where $\mu_{s},\mu_{f}$ are the viscosities of the solid and fluid, $V_{i}$ is the volume of the cell, and $I\left(x,t\right)$ is an indicator function (i.e., $I\left(x,t\right)=1$ indicates solid, and $I\left(x,t\right)=0$ indicates fluid). Material properties and geometric parameters are presented in Table 1. The transport equation of the volume fraction of the solid is

(5) $$\begin{array}{c} \partial_{t}\alpha +\nabla \cdot \left(\alpha u\right)=0 \end{array}$$

**TABLE 1:** Fluid and solid parameters in the present simulation.

Parameter	Symbol	Value/range
Pipe length	L	5.0 mm
Pipe diameter	D	1.0 mm
Fluid density	$\rho_{f}$	1000 kg m^−3^
Fluid viscosity	$\mu_{f}$	1.0 × 10^−5^ Pa s
Solid density	$\rho_{s}$	1000 kg m^−3^
Solid viscosity	$\mu_{s}$	1.0 × 10^−5^–1.0 × 10^−4^ Pa s
Mean velocity of fluid in the pipe	u	50 mm s^−1^ ^a^
Solid shear modulus	G	10–100 Pa
Major radius of torus	R	0.15–0.325 mm
Minor radius of torus	r	0.05–0.125 mm

^a^ The value of blood flow velocities is referenced in the previous report [22].

In addition, the Cauchy stress tensor consists of the pressure and the deviatoric stress tensors of both fluid and solid as well as

(6) $$\begin{array}{c} \sigma =-pI+\left(1-\alpha \right)\sigma_{f}^{\prime }+\alpha \sigma_{s}^{\prime } \end{array}$$

where p is the pressure, I is the unit tensor, $\sigma_{f}^{\prime },\sigma_{s}^{\prime }$ are the deviatoric stress tensors of the fluid and solid, and $A^{\prime }\left(=A-\frac{1}{3}\mathrm{tr}\left(A\right)I\right)$ is a deviatoric tensor. The deviatoric stress tensor of the fluid is composed of viscous stress described as

(7) $$\begin{array}{c} \sigma_{f}^{\prime }=2\mu_{f}D^{\prime } \end{array}$$

where $D\left(=\frac{1}{2}\left(\nabla u+\nabla u^{T}\right)\right)$ is the shear rate tensor. The deviatoric stress tensor of the solid is composed of viscous stress and elastic stress described as

(8) $$\begin{array}{c} \sigma_{s}^{\prime }=2\mu_{s}D^{\prime }+GB^{\prime } \end{array}$$

where G is the shear modulus of the solid, and $B^{\prime }$ is the deviatoric tensor of the left Cauchy‐Green deformation tensor which represents the deformation state inside the solid. The second term in Equation (8) represents the contribution of the Cauchy stress of the visco‐hyperelastic solid. We assumed the solid to be a Neo‐Hookean visco‐hyperelastic material due to the rheological characteristics of alginate hydrogels [23]. The initial value of B in the solid is the unit tensor (I). The transport equation of the left Cauchy‐Green deformation tensor is obtained by transforming its material derivatives as

(9) $$\begin{array}{c} \partial_{t}B+\nabla \cdot \left(\mathrm{uB}\right)=L\cdot B+B\cdot L^{T} \end{array}$$

where $L\;\left(={\left(\nabla u\right)}^{T}\right)$ is the velocity gradient tensor. To avoid numerical instability of the divergence term, we calculated the Equation (9) only around the solid with volume fraction as $\tilde{B}=\alpha^{\beta }B$. To satisfy the incompressibility of the solid, β should equal 1/2. Therefore, the transport Equation (9) is reformulated as

(10) $$\begin{array}{c} \partial_{t}\tilde{B}+\nabla \cdot \left(u\tilde{B}\right)=L\cdot \tilde{B}+\tilde{B}\cdot L^{T} \end{array}$$

Then, the deviatoric elastic Cauchy stress tensor is described as follows:

(11) $$\begin{array}{c} \alpha \sigma_{s}^{\prime }=2\alpha \mu_{s}D^{\prime }+\alpha^{1/2}G\tilde{B} \end{array}$$

To summarize these equations, the equation of momentum conservation [2] is reformulated as follows:

(12) $$\begin{array}{c} \partial_{t}u+\left(u\cdot \nabla \right)u=-\frac{1}{\rho }\nabla p_{\mathrm{rgh}}+2\left\{\left(1-\alpha \right)\nu_{f}+\alpha \nu_{s}\right\}\left(\nabla \cdot D^{\prime }\right)+\frac{\alpha^{1/2}}{\rho }G\left(\nabla \cdot \tilde{B}\right)+S \end{array}$$

where $\nu_{f},\nu_{s}\;\left(=\mu_{f}/\rho ,\mu_{s}/\rho \right)$ are kinematic viscosities of the fluid and solid, respectively, $p_{\mathrm{rgh}}\;\left(=p-\rho g\cdot x\right)$ is an alternative pressure obtained by subtracting the hydrostatic pressure from the pressure, and S is a source term for the periodic boundary condition. The momentum conservation in Equation (12) describes in one equation using volume fraction of the solid so that the law of action/reaction holds everywhere. By Boussinesq approximation in the isothermal condition, the alternative pressure is used in calculations instead of the pressure in the multiphase solver within OpenFOAM [18].

### 2.3 Simulation Methods

In this study, we implemented a full Eulerian FSI solver in OpenFOAM v2212, an open‐source CFD toolbox using the finite volume method on unstructured meshes. The original solver, *interIsoFoam*, based on the algebraic VOF solver *interFoam* was introduced by Roenby et al. [24]. This solver employs geometric reconstruction methods called *isoAdvector* to ensure accurate interface evolution and moderate spurious currents. Among these methods, this solver uses *plicRDF*, an iterative method for estimating the interface center and normal vector within interface cells. The piecewise linear interface construction (PLIC) scheme is one of the geometric reconstruction methods introducing isosurface in an interfacial cell. This method utilizes a reconstruction distance function (RDF, Ψ) to compute a weighted average of distances to the interfaces within the cell and its neighboring points [25]. The unit normal vector in the interface cell, $n_{s}$, is finally obtained by the gradient of RDF as follows:

(13) $$\begin{array}{c} n_{s}=\frac{\nabla \Psi }{\left|\nabla \Psi \right|} \end{array}$$

**SCHEME 1:**
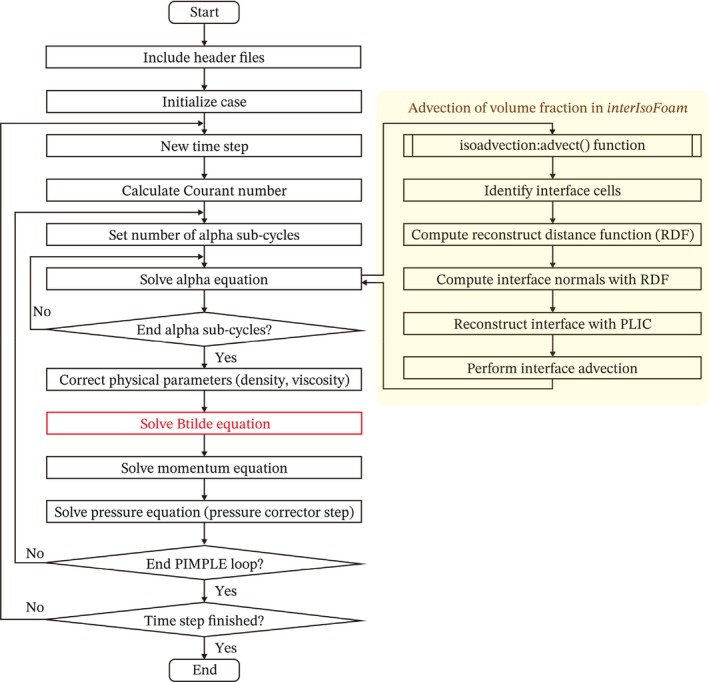
Flow diagram of the solver algorithm with *plicRDF* reconstruction scheme. The solver is based on the PIMPLE algorithm and incorporates the transport equation of the corrected left Cauchy‐Green deformation tensor $\tilde{B}$ in Equation (10) into the original *interIsoFoam* framework. The volume fraction field is advected using the interface velocity computed via the reconstruction distance function (RDF), which enhances interface accuracy and stability.

This method improved the convergence of iterative errors of the normal vector and the curvature by using the normal vector of the previous time step as an initial value [25]. The solution algorithm in the present study is shown in Scheme 1.

We added the elastic stress term in the third term on the right‐hand side of Equation (12) in *interIsoFoam* and applied the Crank–Nicolson scheme for the time evolution of the corrected left Cauchy‐Green deformation tensor in Equation (10). The advection term of $\tilde{B}$ is spatially discretized by the Weighted Essentially Nonoscillatory (WENO) scheme [26, 27]. The time evolution of $\tilde{B}$ in Equation (10) is computed between advection of the interface in Equation (5) and solving momentum Equation (12). At each time step, if the value of the volume fraction is less than the minimum value, $\alpha_{\min}$, it is set to 0, and $\tilde{B}$ is converted to a zero tensor (0) or a unit tensor (I) depending on the simulation. The minimum volume fraction is set to 0.1 in accordance with the previous report [17]. The coupling of velocity and pressure fields at each time step is achieved through the PIMPLE algorithm which integrates the Pressure Implicit Split Operator (PISO) and Semi‐Implicit Methods for Pressure Linked Equations (SIMPLE) algorithms [18]. To prevent checkerboard instabilities in pressure and velocity fields due to the use of a collocated grid, OpenFOAM employs Rhie‐Chow interpolation [28].

All parameters in the present simulation are nondimensionalized by the pipe diameter (D), the fluid density ($\rho_{f}$), and the mean velocity of fluid (u). The dimensionless parameters used in the simulation are shown in Table 2. In *fvSchemes* dictionary, the temporal term of the corrected left Cauchy‐Green deformation tensor is discretized using an implicit Crank–Nicolson scheme, while the other temporal terms are discretized using an implicit Euler scheme. Spatial discretization schemes for the advection term are performed using a second‐order implicit upwind scheme with a gradient limiter for the velocity field in equation [12], a second‐order explicit linear scheme for the divergence of the viscous and elastic stress terms in Equation (12), and an implicit WENO scheme for the advection term of the corrected left Cauchy‐Green deformation tensor in Equation (10). The order of the polynomial in the WENO scheme is set to three, which determines the degree of freedom of the polynomial basis function in the WENO reconstruction method [26]. In *fvSolution* dictionary, the pressure equation is treated implicitly by the Preconditioned Conjugate Gradient (PCG) solver using the Diagonal‐based Incomplete Cholesky (DIC) preconditioner, while the momentum equation and the transport equation of the corrected left Cauchy‐Green deformation tensor are treated by a smoothsolver with GaussSeidel smoother in OpenFOAM. In the *fvOptions* dictionary, we employed the *patchMeanVelocityForce* utility to generate a stable Hagen–Poiseuille flow within a pipe with periodic boundaries. This utility introduces a pseudo‐pressure gradient as a uniform source term in the momentum equation, applied to all cells in the domain. The magnitude of this gradient is dynamically adjusted to maintain a mean inlet velocity of 0.5.

**TABLE 2:** Dimensionless parameters in the present simulation.

Parameter	Symbol	Value/range
Dimensionless time	$t^{*}=D/2\bar{u}$	0–20
Reynolds number	Re=ρfu¯D/μf	50
Courant number	$\mathrm{Co}=2\bar{u}\Delta t/\Delta x$	<0.1 ^a^

^a^ Δt is automatically corrected during computation with satisfying the Courant–Friedrichs–Lewy (CFL) condition.

In *controlDict* dictionary, the initial time step is set to 10^−4^, but the time step size is automatically adjusted in OpenFOAM to satisfy the Courant–Friedrichs–Lewy (CFL) condition. The conditions for calculation stability for the viscous and elastic stress terms are described as:

(14) $$\begin{array}{c} 3\nu_{s}\frac{\Delta t}{{\left(\Delta x\right)}^{2}}<1 \end{array}$$

(15) $$\begin{array}{c} \sqrt{\frac{G}{\rho }}\frac{\Delta t}{\Delta x}<1 \end{array}$$

where Δt is the time step, and Δx is the step size. In the present simulation, we set the Courant number to 0.1.

**FIGURE 2:**
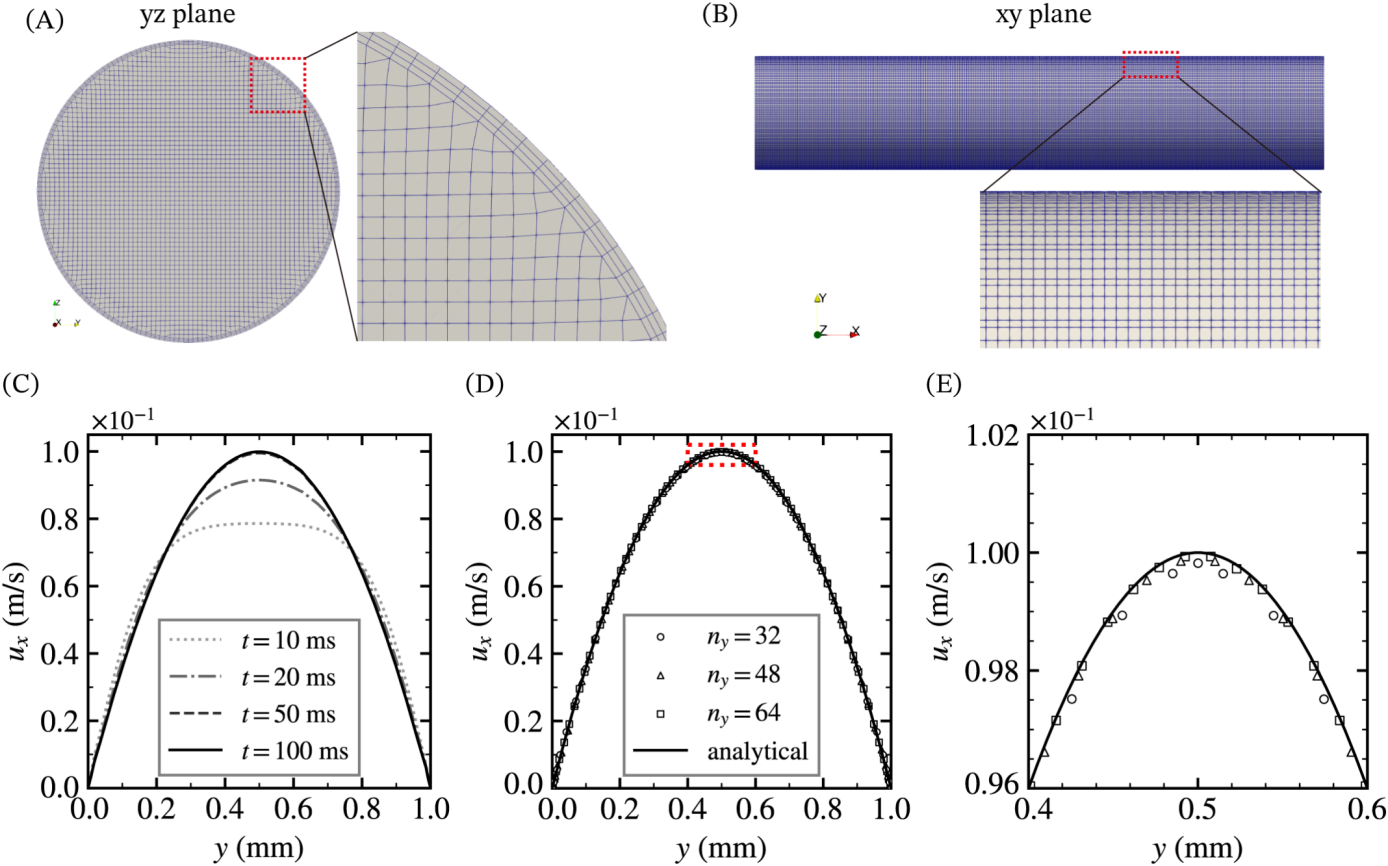
The mesh study in the computational domain. (A, B) The mesh structure of the cylindrical pipe from the (A) yz‐plane and (B) xy‐plane. (C) Time course changes of velocity distribution along a line located at x=2.5, z=0.5 mm. (D, E) Velocity distribution at 100 ms. Panel (E) presents an enlarged view of the region enclosed by the red dotted line in Panel (D). The solid line represents the analytical solution for Hagen–Poiseuille flow. $n_{y}$ denotes the number of hexagonal mesh elements in the y‐direction of the rectangular mesh specified by blockMesh utility. The number of cells for the open circle, triangle, and square markers are 170,880, 528,000, and 1,195,520, respectively.

The mesh in OpenFOAM was constructed using several utilities. In order to use the periodic boundary condition with the unstructured mesh, the mesh structure needs to align on the two corresponding boundaries. First, the original pipe with a length of *L*/2 was designed using FreeCAD software [29], and fine triangular mesh data on the pipe surface were exported via an STL file. Next, *blockMesh* utility created the initial hexagonal mesh covering the entire domain of the pipe. Then, *snappyHexMesh* utility removed unnecessary cells, refined the cell structure along the STL data, and added boundary layers of hexagonal mesh on the wall. Other utilities (*mirrorMesh, topoSet, createPatch*) copied the mesh to the opposite side and renamed the copied boundary as the outlet. The cross‐section of the pipe mesh is shown in Figure 2A,B. The mesh structure was based on a 320 × 64 × 64 rectangular solid, with about 12 cells in the diameter of a torus cross‐sectional circle.

### 2.4 Verification of the Numerical Results

We used three types of mesh to check the convergence and accuracy of velocity field. The boundary conditions at the inlet and outlet are periodic (*cyclic*) for all quantities, which are identical at corresponding cells on the inlet and the outlet. In addition, the sides of the pipe have wall boundary conditions which applies the Neumann condition for *p*_rgh_ and the Dirichlet conditions for the velocity vector (u=0), the volume fraction (α=0), and the corrected left Cauchy‐Green deformation tensor ($\tilde{B}=I$). The initial values of each parameter are $p_{\mathrm{rgh}}=0,u=0,\alpha =0$, and $\tilde{B}=I$, respectively. Figure 2C shows the temporal change of the velocity distribution at the centerline of the cylinder for 100 ms. Figure 2D,E shows the velocity distribution along the centerline at *x* = 25 using different mesh sizes, and the analytical velocity distribution described as follows:

(16) $$\begin{array}{c} u_{x}\left(y\right)=8\bar{u}y\left(1-y\right)\;\left(0\leq y\leq 1\right) \end{array}$$

The accuracy of the velocity fields is given by the *L*_2_ norm described as

(17) $$\begin{array}{c} \left\|L_{2}\right\|\left(u_{x}\right)={\left\{\frac{1}{N}\sum_{i}{\left|u_{\mathrm{xi}}-u_{x}\left(y_{i}\right)\right|}^{2}\;\right\}}^{\frac{1}{2}} \end{array}$$

where $u_{\mathrm{xi}}$ is the *x*‐component of the velocity vector at cell *i*, and $u_{x}\left(y_{i}\right)$ is the analytical solution of Equation (16) at the *y*‐coordinate of the cell *i*. The *L*_2_ norms for each mesh are $1.2\times 10^{-5},0.47\times 10^{-5},0.15\times 10^{-5}$, respectively. The finest mesh shows good agreement with the analytical solution, so we used this mesh for the following simulations.

**FIGURE 3:**
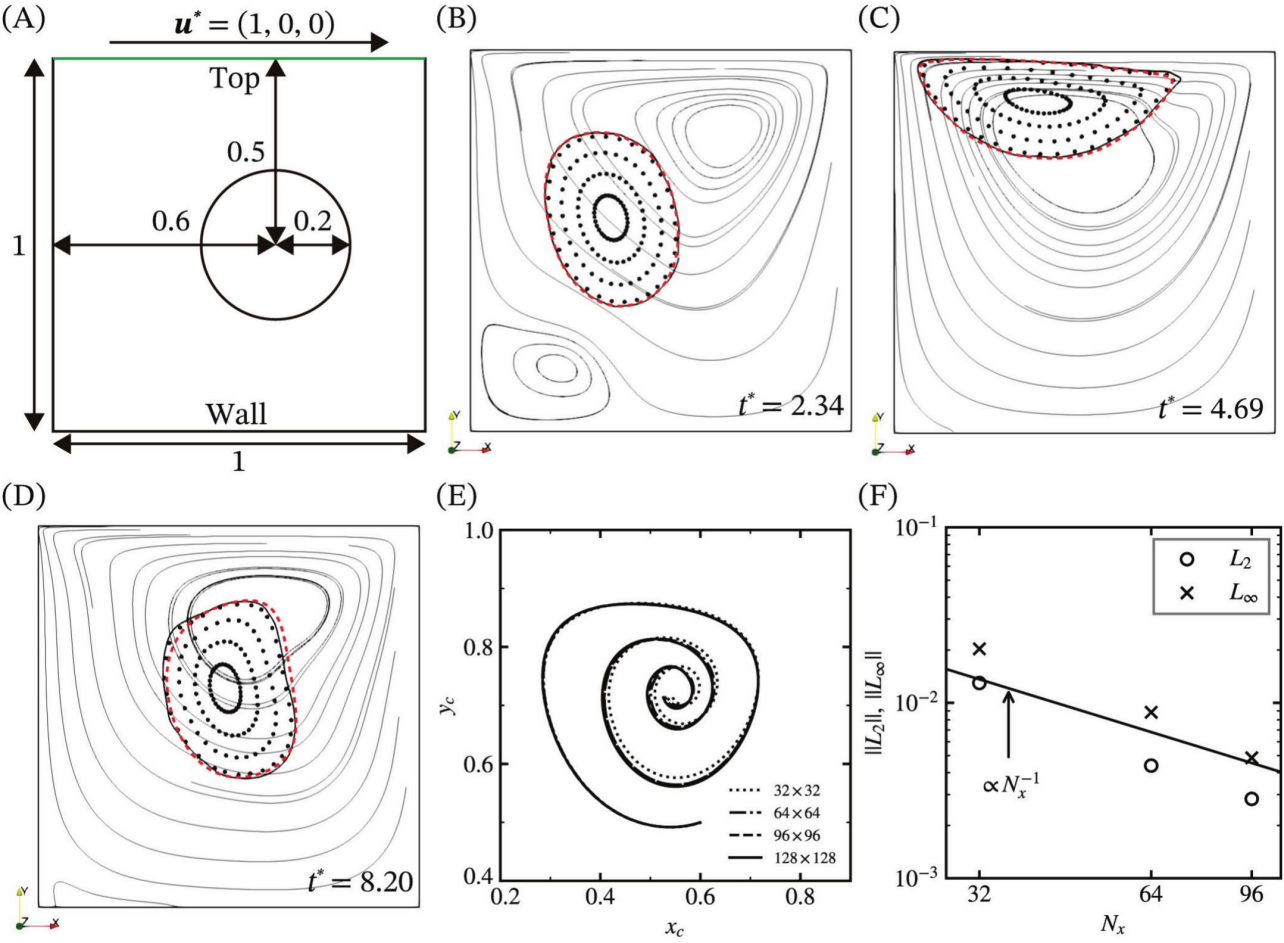
Comparison of the solid deformation in the lid‐driven flow with the simulation result [17, 30]. (A) Schematic setup of the simulation. (B–D) Snapshots of the solid contour, Lagrangian tracking particles, and the streamlines. The red dashed line represents the results of Zhao et al. [30] The bold solid line represents the isoline of the volume fraction at α=0.5, and the narrow solid line indicates the streamlines with a 128 × 128 mesh. (E) Trajectory of the solid centroid in the lid‐driven flow over the time range $t^{*}\in \left[0,20\right]$ for various numbers of grid points. (F) The errors of the solid centroid in $L_{2}$ norm and $L_{\infty }$ norm versus the number of grid points ($N_{x}$) in the lid‐driven flow.

Next, we numerically verified the solver by comparing the solid deformation in a two‐dimensional lid‐driven cavity flow with previous reports from Zhao et al. [30] and Sugiyama et al. [17]. Briefly, the schematic illustration of the simulation is shown in Figure 3A. The cavity has dimensions of 1 × 1, with the top boundary named “top,” and the other boundaries named “wall.” The visco‐hyperelastic solid is centered at (0.6, 0.5) with a radius of 0.2, $\rho =1,\mu_{s}=\mu_{f}=0.01$, and *G* = 0.05. Wall boundaries are treated as Wall boundary conditions as described in the previous subsection. At the top boundary, the wall moves in the *x*‐direction with $u_{x}=1$. Initial conditions of velocity are u=0, p=0, $\tilde{B}=0$. In this case, we apply the corrected value of $\tilde{B}$ as a zero tensor. The nondimensional simulation time ($t^{*}$) is from 0 to 20.

Figure 3B–D shows the solid isolines and Lagrangian tracking particles at three time points. Lagrangian tracking particles are located in the visco‐hyperelastic solid and move along the velocity fields. The narrow solid line represents the streamlines, the bold solid line indicates the isoline of the volume fraction at 0.5, and the red dashed line shows the results obtained by Zhao et al. [30]. The shape of the solid agrees with the results of the previous report at the early time points, while the surface of the solid at $t^{*}=8.20$ is slightly different. Figure 3E shows the trajectory of the solid centroid over the time range $t^{*}\in \left[0,20\right]$. We use four types of mesh with different numbers of grid points to check convergence and accuracy. The centroid of the solid, $x_{c}\left(t\right)$, is approximated by

(18) $$\begin{array}{c} x_{c}\left(t\right)\approx \frac{\sum_{i}\alpha \left(x_{i},t\right)x_{i}V_{i}}{\sum_{i}\alpha \left(x_{i},t\right)V_{i}} \end{array}$$

where $x_{i}$ is the position of the cell center, and $V_{i}$ is the cell volume. The trajectory of the centroid without the roughest mesh grid agrees with the finest mesh grid. Figure 3F shows the errors of the centroid trajectory in *L*_2_ and $L_{\infty }$ norms described as follows:

(19) $$\begin{array}{c} \left\|L_{2}\right\|\left(N_{x}\right)={\left\{\frac{1}{t_{\max}^{*}}\sum_{i}{\left|x_{c}\left(t^{*},N_{x}\right)-x_{c}\left(t^{*},N_{x}=128\right)\right|}^{2}\;\right\}}^{\frac{1}{2}} \end{array}$$

(20) $$\begin{array}{c} \left\|L_{\infty }\right\|\left(N_{x}\right)=\max_{t^{*}\in \left[0,t_{\max}^{*}\right]}\left|x_{c}\left(t^{*},N_{x}\right)-x_{c}\left(t^{*},N_{x}=128\right)\right| \end{array}$$

where $t_{\max}^{*}\;\left(=20\right)$ is the maximum time point in this case. The errors of the centroid linearly decrease to the reciprocal number of the grid points, which is related to the spatial discretization of the elastic stress term in Equation (12). The results of both verifications show that 64 grid points are sufficient for FSI analysis in terms of velocity fields and solid deformation.

## 3 Results and Discussion

### 3.1 Flow Behavior of a Torus With a Different Initial Orientation and Position

**FIGURE 4:**
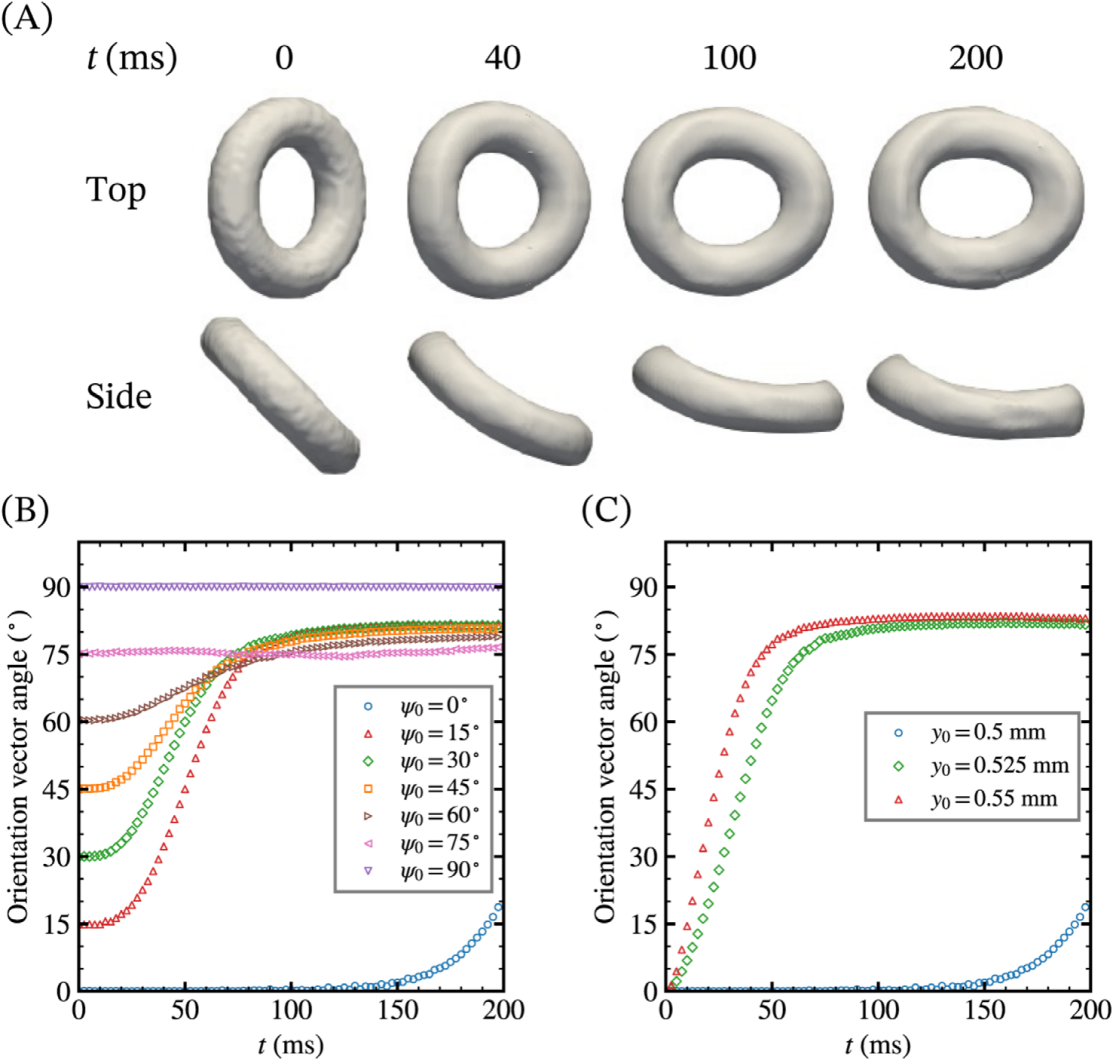
Shape and orientation of a torus. (A) Top and side views of the isosurface of volume fraction of the visco‐hyperelastic torus at α=0.5. The torus rotates and deforms within 200 ms. (B, C) Time course changes of the angle of the orientation vector with different initial orientation ($y_{0}=0.5$ mm) and position ($\psi_{0}=0{}^{\circ}$). The angles of the orientation vector converge with multiple steady states responding to the initial condition.

We first analyze the effect of the initial condition on the orientation of a torus. Figure 4 shows the isosurface of a torus at α=0.5 and the angle of the orientation vector. The simulation time range is set to 200 ms to capture the complete rotation of the torus. Following previous studies [15, 31], the orientation vector of the deformed torus is defined as the eigenvector associated with the smallest eigenvalue of the gyration tensor *G*(*t*), which is formulated as

(21) $$\begin{array}{c} G\left(t\right)=\frac{1}{N}\sum_{i=1}^{N}\left(x_{i}\left(t\right)-x_{c}\left(t\right)\right)⨂\left(x_{i}\left(t\right)-x_{c}\left(t\right)\right) \end{array}$$

where N is the number of points on the isosurface of the torus at α=0.5, $x_{i}$ is the position vector of i‐th point on the isosurface, and $x_{c}$ is the centroid of the torus, respectively. In these cases, the torus rotates along the z‐direction, so its orientation can be determined from the inner product of the orientation vector and a unit vector along the x‐axis. In Figure 4A, the torus initially inclined at 45° rotates to approximately 80° with bending and elongation in the flow direction over time (Movie S1). Additionally, Figure 4B shows that the rotation angles of the torus located at the pipe center converge with different steady states depending on the initial orientation. The horizontal torus ($\psi_{0}=90{}^{\circ}$) does not rotate throughout the simulation due to its plane‐symmetric structure relative to the flow direction (Movie S2). However, the vertical torus ($\psi_{0}=0{}^{\circ}$) does not rotate initially but begins to rotate in the middle of the simulation (Movie S3). This suggests that the vertical torus in Hagen–Poiseuille flow is unstable, and a small asymmetric perturbation can induce rotation. In addition, the angles of the inclined tori, except at 75°, increase monotonically to around 80° after a short waiting period of 20 ms, while the angle of the torus inclined at 75° slightly fluctuates throughout the simulation. Tori centered away from the pipe center also rotate to around 80°, but without the waiting period as shown in Figure 4C. The waiting period is related to the development of the velocity gradient from the pipe side. Thus, the vertical torus near the wall starts to rotate earlier than the one located at the pipe center.

**FIGURE 5:**
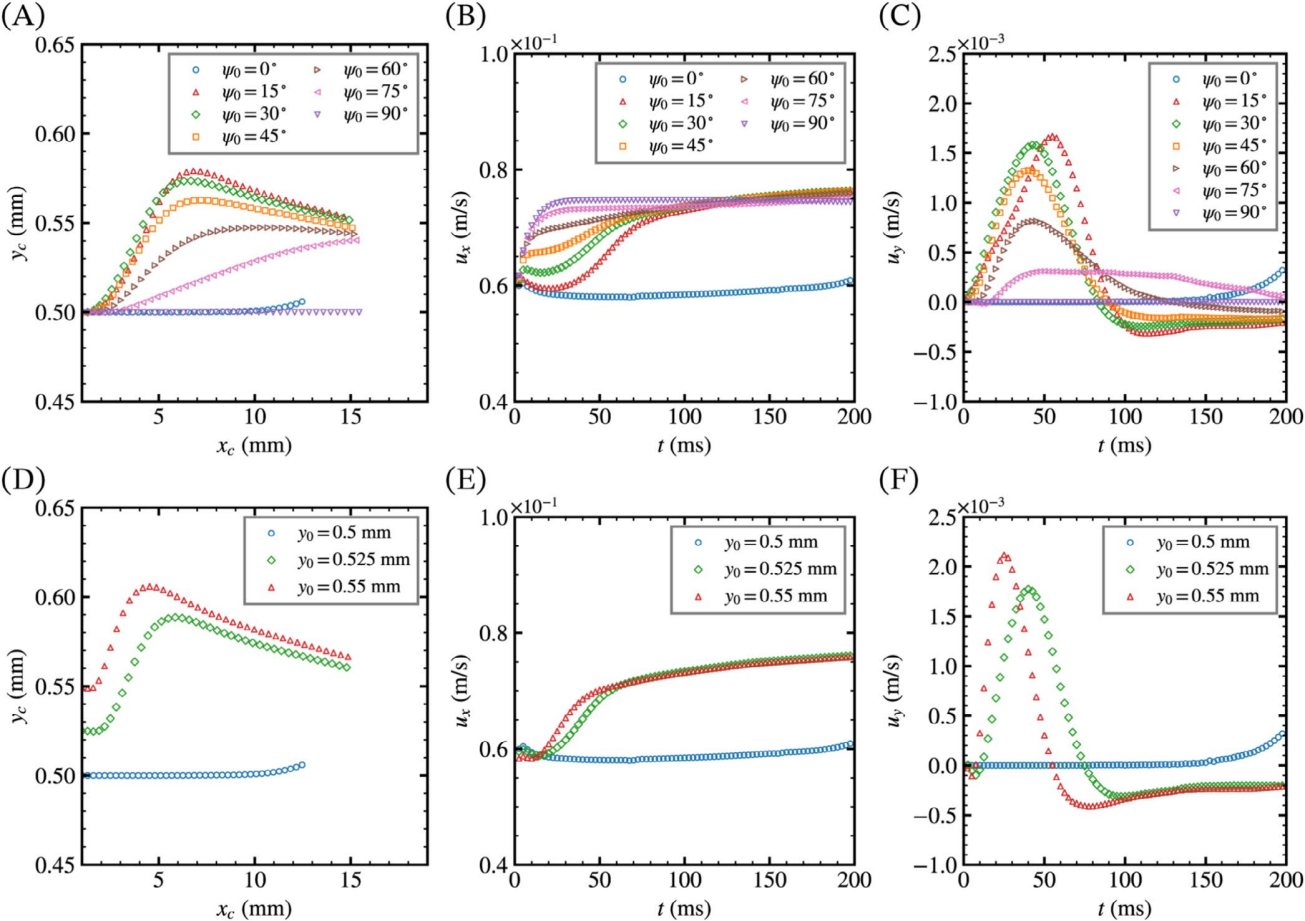
Movement and velocity components of the tori with (A–C) different initial orientations and (D–F) different initial positions. Time course changes of (A, D) the position of the centroid, (B, E) the x‐component of the centroid velocity vector, and (C, F) the y‐component of the centroid velocity vector.

Figure 5 shows the time course changes of the centroid position and velocity components with the difference in the initial orientation and position. The centroid of the torus is approximated by Equation (18) and the centroid velocity $u_{c}$ is also approximated as follows:

(22) $$\begin{array}{c} u_{c}\left(t\right)\approx \frac{\sum_{i}\alpha \left(x_{i},t\right)u\left(x_{i},t\right)V_{i}}{\sum_{i}\alpha \left(x_{i},t\right)V_{i}} \end{array}$$

The y‐coordinate of the centroid of the inclined tori moves away from the pipe center and then converges, whereas that of the vertical or horizontal tori does not change. In the cases of $\psi_{0}$ = 15°, 30°, 45°, $u_{x}$ shows a local maximum at early time points within 10 ms, and $u_{y}$ also shows different local maximum. In contrast, in the cases of $\psi_{0}$ = 60°, 75°, $u_{x}$ does not show a local maximum, and $u_{y}$ decreases relatively slowly. As shown in Figure 5D–F, the tori centered away from the pipe center show similar trends in centroid position and velocity but relatively higher maximum $u_{y}$ compared with those of the inclined tori. It is noted that the maximum $u_{x}$ of the inclined tori is higher than that of the horizontal torus. The horizontal torus without any deformation is assumed to be the fastest since its centroid is located on the centerline of the pipe and the orientation of the major radius is horizontal to the flow direction. In fact, the drag coefficient and its force of the inclined torus minimize at 90° in uniform steady flow [32]. Thus, the effects of solid deformation on the flow of the inclined tori need to be considered.

### 3.2 Deformation Analysis of a Torus

**FIGURE 6:**
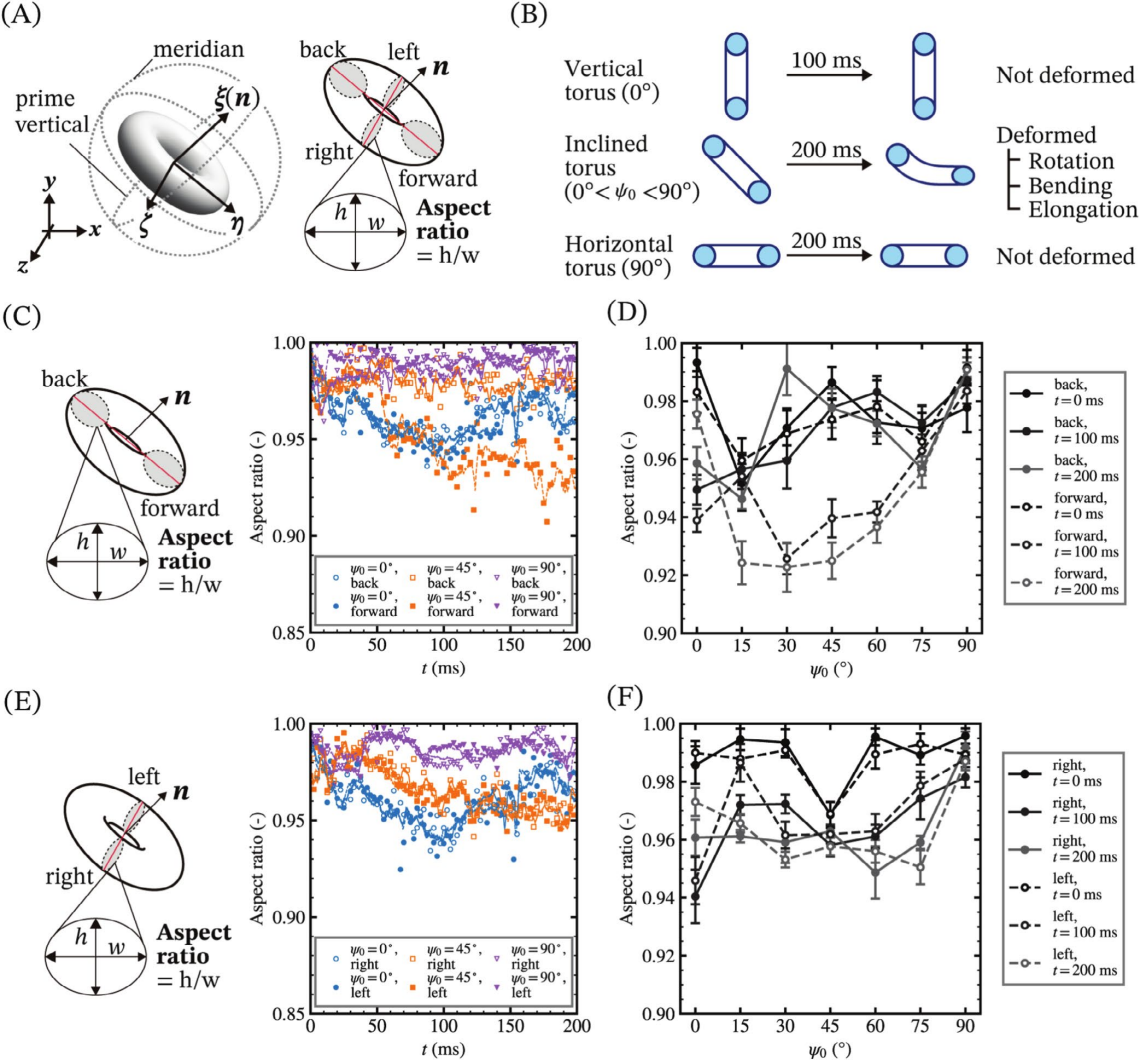
Deformation analysis of the cross‐section of a torus. (A) Schematic illustration of the deformation analysis. The local coordinate axes of a torus (ζ,η,ξ) is defined by transforming the global Cartesian coordinates (x,y,z). Two cross‐sectional planes—the meridian and prime vertical—are used to extract four representative isolines, labeled as forward, back, right, and left. (B) Schematic illustration of the deformation of tori with different initial orientations. (C, E) Schematic illustration and time course changes of the aspect ratios of the isolines on each cross‐section. The aspect ratios of the isolines are obtained via elliptical approximation. The solid and dashed lines represent the moving average of up to 25 data points (Δt=2.5 ms). (D, F) Comparison of aspect ratios at specific time intervals for tori with different initial orientations. Each data point represents the mean aspect ratio over 20 samples (Δt=2 ms), with error bars indicating standard deviation. Notably, the aspect ratios of the forward isolines are reduced compared to those of the back isolines and to the forward isolines of horizontally and vertically oriented tori.

To explore the flow of the deformable torus, we analyze its deformation from several perspectives. First, we focused on the shape of the cross‐section of the torus over time. Figure 6A shows a schematic illustration of the deformation analysis of the cross‐section of the torus. In order to trace the deformation of a rotating torus, we introduce a local coordinate system (ζ,η,ξ) centered at the centroid of the torus. ξ corresponds to the orientation vector (n), ζ corresponds to the unit vector of the z‐axis at the initial time point, and η is obtained from the cross product of the other axes. The same cross‐section at each time step is obtained by rotating ξ from the initial orientation. In the local coordinate system, a circle on the plane formed by η and ζ is named the meridian, and that formed by ξ and ζ is named the prime vertical. The resulting four isolines of the cross‐sections on the meridian and prime vertical planes are named forward, back, right, and left, respectively. Aspect ratios of the isolines are obtained by elliptical fitting using the least squares method with the following equation:

(23) $$\begin{array}{c} \frac{{\left(X\cos\theta +Y\sin\theta \;\right)}^{2}}{h^{2}}+\frac{{\left(-X\sin\theta +Y\cos\theta \right)}^{2}}{w^{2}}=1 \end{array}$$

where X,Y are the local two‐dimensional coordinates, h,w are the height and width of the ellipse, and θ is the rotation angle of the ellipse.

Figure 6B shows the schematic illustration of the deformation of the tori with different initial orientation. The vertical and horizontal tori do not show apparent deformation unless it starts to rotate, while all inclined tori deformed over time. The deformation behaviors are classified into three modes: rotation, bending, and elongation. Figure 6C,E and shows the time course changes of the aspect ratio of the two isolines on each cross‐section. The solid and dashed lines represent moving averages of the aspect ratio with up to 25 time points. Moreover, Figure 6D,F shows the dependency of aspect ratios of the cross‐section of tori with different initial orientation at the specific time range. In the meridian direction, there are no significant differences in the aspect ratios of the forward and back ellipses in the cases of $\psi_{0}$ = 0°, 90°, while the aspect ratio of the forward ellipse decreases over time in the case of $\psi_{0}=45{}^{\circ}$. This indicates that the cross‐section of a torus on the meridian plane deforms anisotropically depending on the local position, and other inclined tori show similar trends as shown in Figures 6D and S1. In the prime vertical direction, the aspect ratios of the right and left ellipses are almost the same, and the aspect ratios of the inclined tori gradually decrease with their elongation in the flow direction as shown in Figure 6C,F. The calculated aspect ratios show vibrations over time, which was caused by some factors such as velocity, aspect ratio of inner and outer radius, and the viscoelasticity of the torus. The vibration modes of an elastic torus with different aspect ratios were numerically evaluated by Zhou et al. [33] The eigenfrequencies of flexural, stretching, and torsional modes depend on the aspect ratio and shear modulus shown in the following equation:

(24) $$\begin{array}{c} K=\mathrm{\kappa r}\sqrt{\rho /G} \end{array}$$

where K is an eigenfrequency parameter and κ is the circular eigenfrequency of the vibration mode of the torus. In the present simulation, the vibration of the torus is attenuated by viscous relaxation and is mainly caused by the fluctuation of the reconstructed isosurface of the torus. In fact, some peaks of the Fourier‐transformed vibration spectra are proportional to $N_{x}u_{x}$ as shown in Figure S2. Thus, finer mesh, more precise surface reconstruction, and less viscous relaxation are needed to understand the vibration modes of the flowing torus.

**FIGURE 7:**
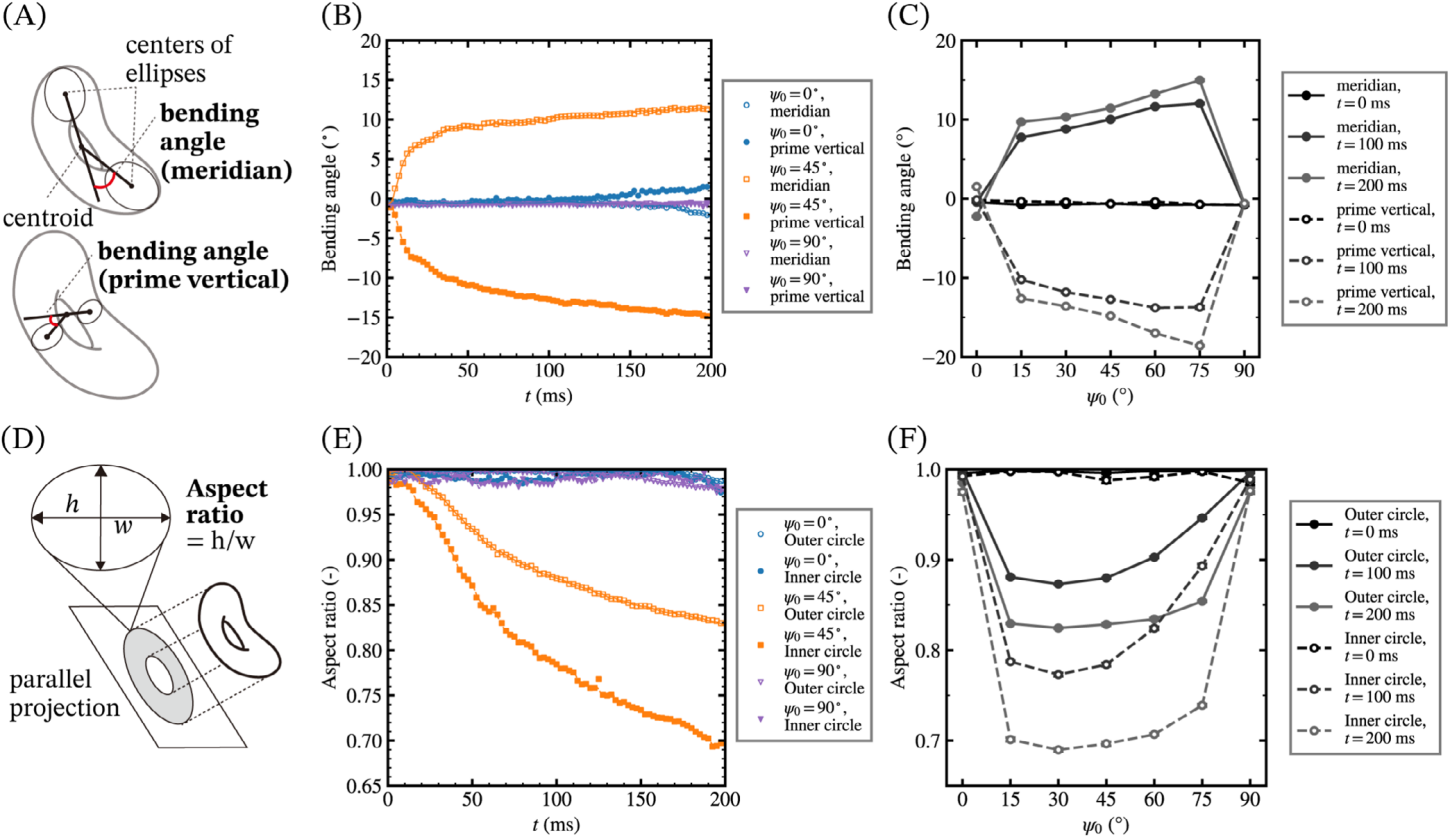
Deformation analysis of the bending and elongation of a torus. (A, D) Schematic illustrations of the analytical methods used to evaluate (A) bending angles and (D) aspect ratio of parallel projected circles of a torus. Bending angles are calculated on each cross‐section using the inner product of vectors connecting the centroid to the centers of the fitted ellipses. The projected circles are obtained by parallel projection of the torus isosurface onto the plane defined by the local coordinates (ζ,η **)**. (B, E) Time course changes in (B) bending angles and (E) aspect ratios of the inner and outer projected circles. The solid and dashed lines represent the moving average of up to 25 data points (Δt=2.5 ms). (C, F) Influence of initial orientation on (C) bending angles and (F) aspect ratios at a specific time interval. Each data point represents the mean aspect ratio over 20 samples (Δt=2 ms), with error bars indicating standard deviation. Inclined tori exhibit a larger amplitude of bending angles and lower aspect ratios compared to those of horizontal and vertical tori.

Next, we focused on the bending and elongation behaviors of the deformed torus over time. Figure 7A shows the schematic illustration of deformation analysis of bending angles. The bending angle of the torus on each cross‐section is determined by the inner product of the two vectors between the centroid and each center of the ellipses. The direction toward ξ set to be plus, so the bending angle of prime vertical direction are minus value. Figure 7B,C show the time course changes of the bending angles for the meridian and prime vertical directions and the dependency of the initial orientation on them. The bending angles of the inclined tori gradually increase, and the angles in the prime vertical direction are larger than those in the meridian direction due to the elongation of the tori in the direction. It is noted that the bending of the torus initially set at 45° occurs within 20 ms, which is faster than the start of the rotation shown in Figure 4B. Moreover, the maximum bending angle of inclined tori at *t* = 200 ms correlates with the initial inclination angle as shown in Figures 7C and S3.

In addition, Figure 7D shows the schematic illustration of deformation analysis of aspect ratios of aspect ratio of projected circles of a torus. Aspect ratios of the inner and outer circles are obtained by the parallel projection of the isosurface of the torus onto a plane perpendicular to ξ and the aspect ratio would be decreased significantly by the elongation of the torus. Figure 7E,F shows the time course changes of the aspect ratio of the two circles and the dependency of the initial orientation on them. The aspect ratios of the inner and outer circles of vertically and horizontally oriented tori hardly decrease, but the inclined tori show a larger decrease in the aspect ratio. As shown in Figure 6B, the deformation processes of the inclined torus can be classified into three steps: bending, rotation, and elongation. The beginning and interval of these processes differ based on the initial position and orientation, and the torus initially inclined at 45° shows apparent differences in each phase. The torus starts to bend immediately with acceleration, and the bending is almost completed within 60 ms. After the initial bending in 20 ms, the torus rotates due to the velocity gradient from 20 to 80 ms, and the inclination of the aspect ratio in Figure 7E decreases due to the reduction in the velocity gradient by the rotation. In other words, highly inclined torus ($\psi_{0}>45{}^{\circ}$) do not show an apparent inclination change in the aspect ratio of the projected circles because they take longer to rotate or bend than slightly inclined tori ($\psi_{0}\leq 45{}^{\circ}$) as shown in Figure S3.

**FIGURE 8:**
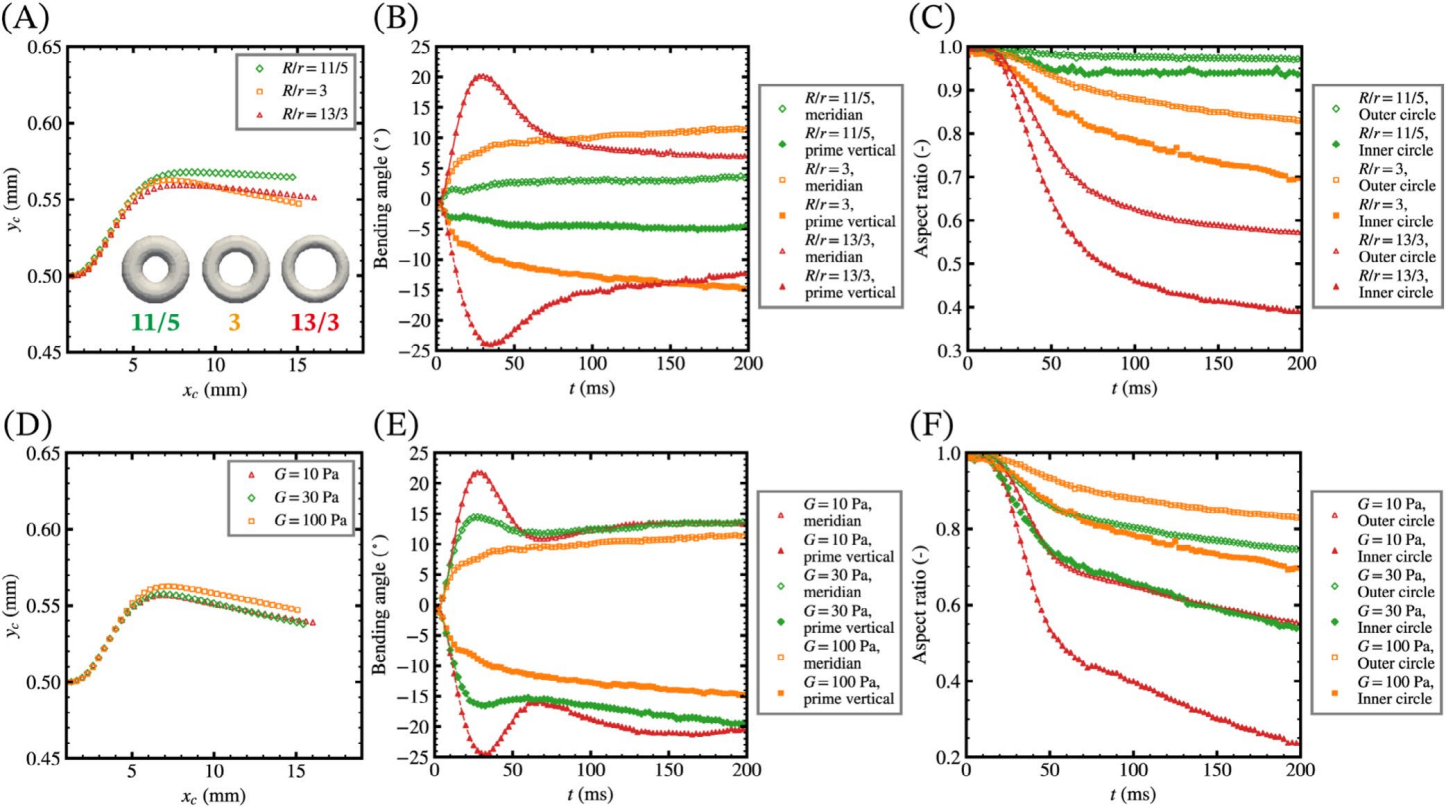
The effects of (A–C) the aspect ratio of the major and minor radii and (D–F) the shear modulus of a torus. Time course changes of (A, D) the position of the centroid, (B, E) the bending angle on each cross‐section, and (C, F) the aspect ratios of the inner and outer circles of the projected circles. The solid and dashed lines represent the moving average of up to 25 data points (Δt=2.5 ms). Narrower and softer tori exhibit greater deformation, as reflected in both bending angles and aspect ratios.

Next, we evaluate the effects of the aspect ratio of the major and minor radii (R/r) on the deformation of the torus initially inclined at 45°. We use a thicker torus (R/r=11/5) and a narrower torus (R/r=13/3) with the same sum of the radii (r+R=0.4 mm). Figure 8 shows the time course changes of the centroid position, the aspect ratios of the two ellipses and two circles, and the bending angle. The centroid of a torus up to 80 ms tends to be closer to the center of the pipe as the aspect ratio increases, and thereafter, the centroid of the original torus (R/r=3) is closest to the center. The narrowest torus shows the largest deformation in the aspect ratios of each circle of the projected circles, and the bending angle shows overshoot and undershoot in the meridian and prime vertical directions, respectively. In addition, we evaluated the effects of the shear modulus on the deformation of the inclined torus and calculated the deformation for softer tori (G=10,30 Pa). The softest torus shows the same trends as the narrowest torus as shown in Figure 8, which indicates that the narrower and softer tori are highly elongated by shear stress. The effects of the viscosity of the torus are also evaluated, showing that a more viscous torus moves toward the wall and slightly suppresses deformation (Figure S4).

The equilibrium position and shape of deformable materials in Hagen–Poiseuille flow are still challenging to estimate due to the various forces acting on them. The equilibrium position is generally determined by the balance of lift forces such as net inertial lift force [34], deformability‐induced lift force [35], Saffman lift force [36, 37], and rotation‐induced lift force [38]. Net inertial lift force is composed of shear gradient‐induced lift force and wall‐induced lift force, and these lift forces primarily attribute to the equilibrium position of rigid particles [34, 39]. Shear gradient‐induced lift force is caused by the parabolic velocity distribution and directs toward the wall, while wall‐induced lift force is caused by the pressure increase near the wall and directs toward the pipe center. Helmy and Barthes‐Biesel previously reported the migration effects of the deformation of a spherical capsule in an unbounded parabolic flow [35]. The deformation of the capsules yields a lift force toward the centerline without the effect of wall‐induced lift force. Saffman and rotation‐induced lift forces act on particles when the particle Reynolds number is small (Rep≪1), and when particles rotate at high angular velocity, respectively. Therefore, the effects of these lift forces are negligible in the present simulation. Di Carlo and coworkers separated anisotropic microparticles with different sizes, shapes, and aspect ratios using a microfluidic device [40, 41]. The authors showed that the equilibrium radial position from the center of the pipe linearly increased with the rotational diameter of the anisotropic microparticles. We also evaluate the effects of the size of tori with the same aspect ratio, and the centroid of a smaller torus at t=200 ms moves further away from the centerline (Figure S5). The relationship between the aspect ratio and the radial position should take into account the increase in the rotation radius due to elongation. However, almost all cases in the present simulation do not completely reach the equilibrium position, so further calculations and parametric studies are needed.

### 3.3 Fluid Behavior and Vortex Appearance

**FIGURE 9:**
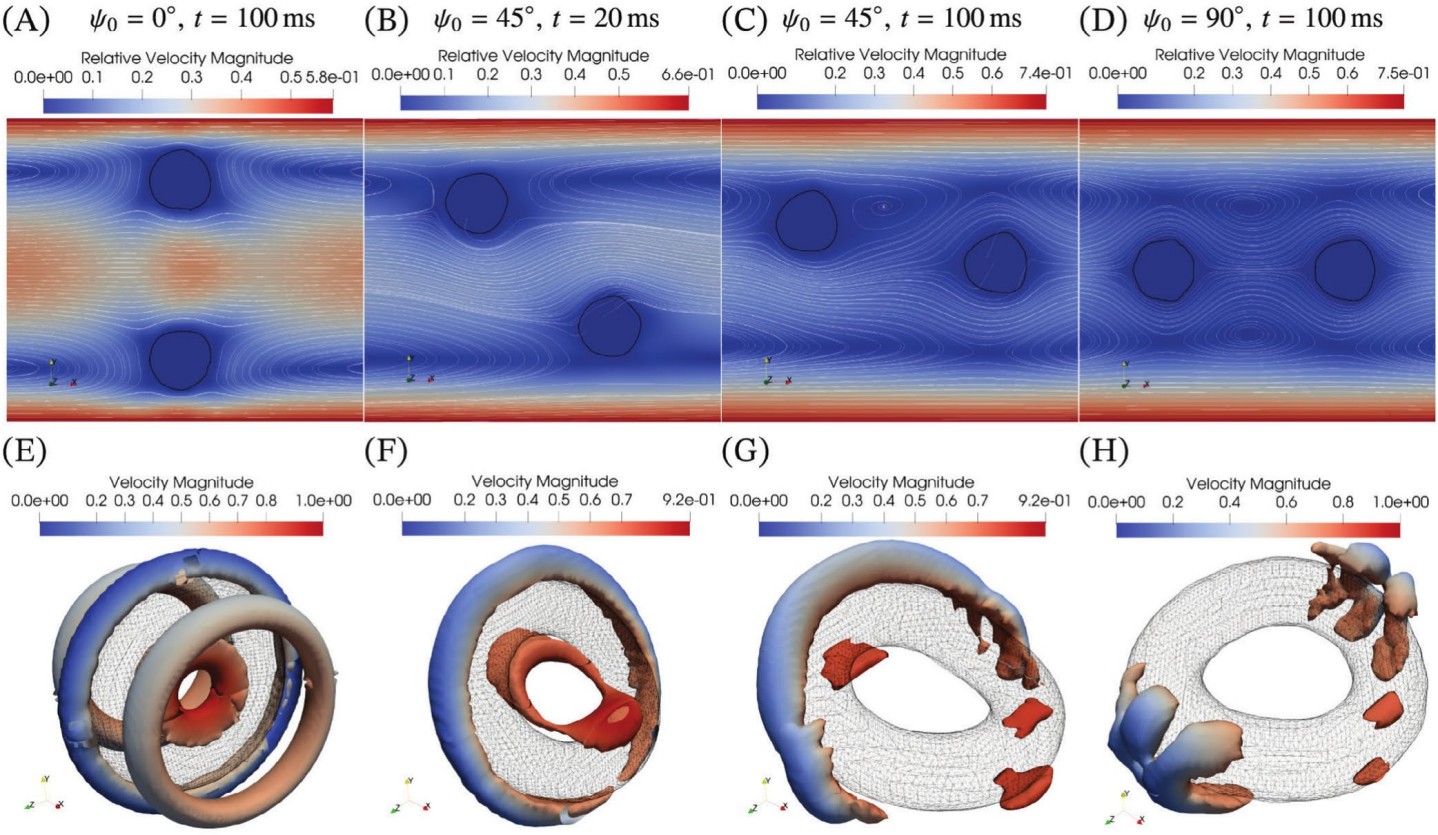
Vortex visualization with relative velocity on a cross‐section and Q‐criterion. Q‐criterion is the second invariant of the velocity gradient tensor [42]. (A–D) Streamlines and colormap of the relative velocity to the centroid velocity on the cross‐section (z=0.5) at each time point (t=100 ms for $\psi_{0}=0{}^{\circ}$ and 90°; t=20 ms and 100 ms for $\psi_{0}=45{}^{\circ}$). (E–H) Vortex visualization by Q‐criterion at each time point. A torus is depicted in the black wireframe, and the color of the isosurface of Q‐criterion at 0.5 shows the fluid velocity. The fluid passes through the hole of the vertical and inclined tori with high velocity, and vortices appear around the torus.

Next, we analyze the fluid flow around the torus using relative velocity and Q‐criterion. Q‐criterion is the second invariant of the velocity gradient tensor proposed by Hunt et al. [42] and is generally used for vortex visualization, which is defined as follows:

(25) $$\begin{array}{c} Q=\frac{1}{2}\left({\left|\Omega \right|}^{2}-{\left|S\right|}^{2}\right) \end{array}$$

where Ω is the vorticity tensor, and S is the deformation rate tensor. Figure 9 shows the absolute relative velocity to the centroid velocity ($u_{c}$) and the isosurface of Q‐criterion at 0.5 around a torus presented as a wireframe. The black solid lines are the isolines of the cross‐section of the torus at α=0.5, and the white solid lines are the streamlines. The fluid passes through the hole of the vertical torus with high velocity, and vortices appear at the forward and back sides of the torus. The isosurface of the Q‐criterion also shows these vortices and additional vortices inside the hole and outside the torus as shown in Figure 9E. Although the fluid passes through the hole of the inclined torus at 45°, the vortices at the forward and back sides disappear. After the deformation, half of the streamlines pass underneath the torus, and the relative velocity to the fluid passing through the hole decreases. Moreover, vortices around the torus gradually decrease over time as shown in Figure 9F,G, which implies that the relative velocity around the torus decreases and the distance between the torus surface and the pipe wall increases. In the case of the horizontal torus, fluid does not pass through the hole, and the streamlines show plane‐symmetric vortices around the torus. The isosurface of the Q‐criterion shows only small vortices in the meridian direction but larger vortices on the side of the prime vertical direction.

**FIGURE 10:**
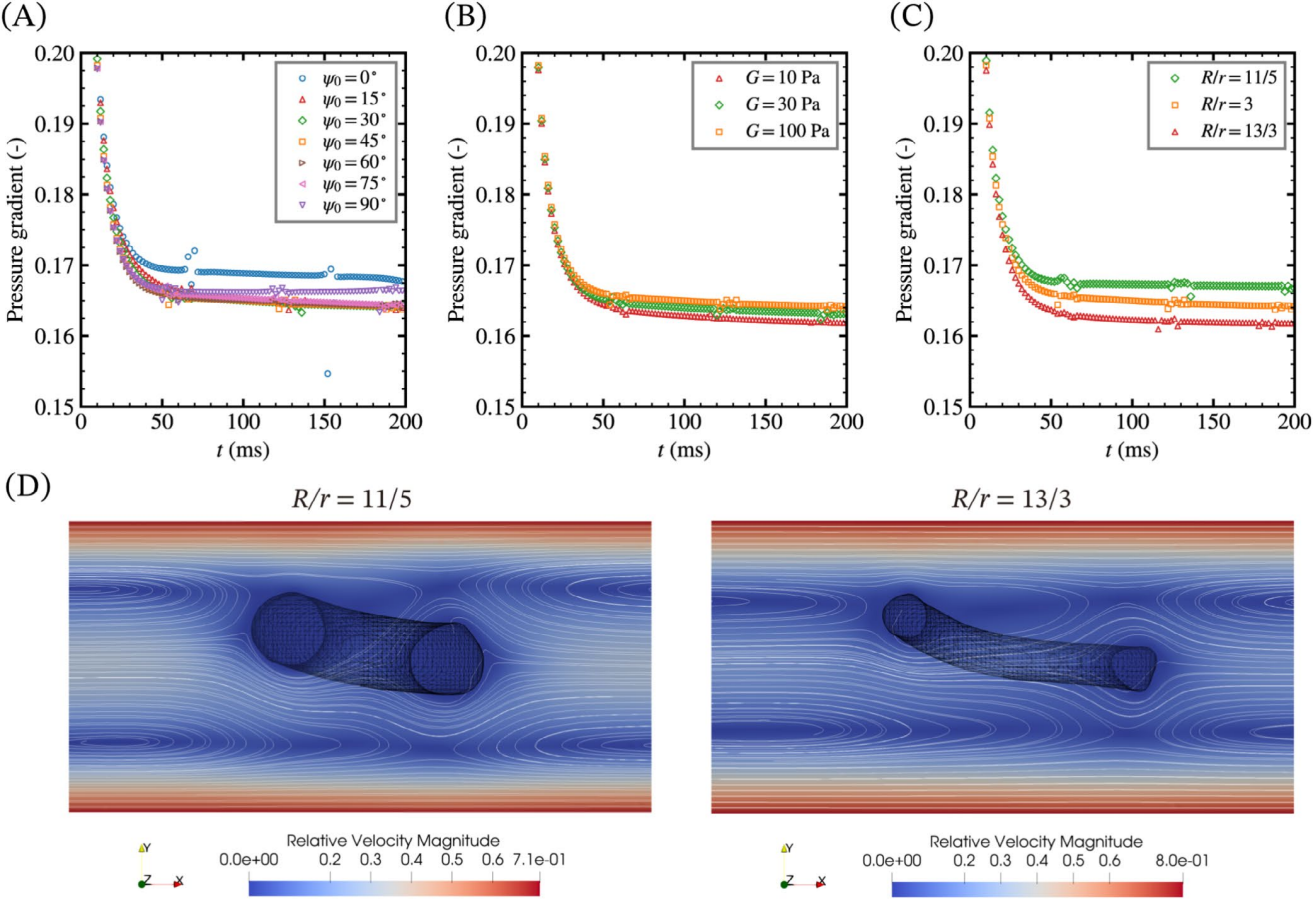
Time course changes of the pressure gradient with the differences in (A) the initial orientation, (B) the aspect ratio of the major and minor radii, and (C) the shear modulus of a torus. The pressure gradient is given in the equation of momentum conservation as a source term (S) to maintain the flow rate at the inlet. The disturbance in the data is caused by the correction of the flow rate during the passage of a torus at the inlet. (D) Relative velocity to the centroid velocity and streamlines with the difference in the aspect ratio of the major and minor radii of a torus. Inclined, softer, and narrower tori show lower pressure gradient compared to the horizontal/vertical tori.

Figure 10 shows the pressure gradient given in the equation of momentum conservation [12] as a source term (S). In this simulation, the pressure gradient is automatically corrected to maintain the flow rate at the inlet, and the product of the pressure gradient and the length of the pipe is equivalent to the pressure drop in the system. This causes fluctuations in the pressure gradient when the torus passes through the boundary, but these fluctuations would not affect the pressure gradient after the passage. The vertical torus shows the highest pressure gradient at 200 ms, while the inclined tori show a relatively lower pressure gradient than the horizontal torus (Figure 10A), which supports the increase in the centroid velocity. Additionally, the pressure gradient for the horizontal torus reaches a steady state, while those for the inclined tori gradually decrease. Figure 10B,C shows that a softer torus and a larger aspect ratio of the major and minor radii decrease the pressure gradient, and the tori in both cases show the largest elongation.

In the present study based on laminar flow, the relative velocity between the torus and the pipe wall primarily causes vortices. This is because the surface of the torus near the wall moves faster than the surrounding flow, generating a relatively large shear rate in the fluid. Such generated vortices result in energy dissipation in the fluid and an increase in the pressure gradient. When a torus is in translational or rotational motion, changes in the distance to the wall decrease the pressure gradient, and the effect would be maximized when the torus is centered on the centerline and inclined at 90°. However, bending and elongation of the torus can increase the distance to the wall, leading to a further decrease in the pressure gradient and an increase in the centroid velocity. Consequently, the horizontal torus hardly deforms due to the symmetric velocity gradient deformation, while the inclined tori gradually deform and reduce the pressure gradient. In comparing the aspect ratios, the overview of the streamlines is almost the same, but the radial length of the narrower torus is shortened by elongation as shown in Figure 10D. Therefore, the deformable torus with bending, rotation, and elongation exhibits different steady states depending on its initial orientation, initial position, structural parameters, and physical properties.

### 3.4 Evaluation of the Embolization in Stenotic Pipe

Finally, we evaluate the embolization behavior in the stenotic pipe with different shapes of microparticles. The length of the stenotic pipe is 6 mm, and the radii of the pipe inlet and the middle of the pipe are 1 and 0.5 mm, respectively. The shapes of the microparticles are torus, disk, sphere, and ellipsoid with the same surface area, and the shape parameters are shown in Table 3. To fix the shape parameters, the ratio of the height to the radius of the disk is two, and the length of the long axis of the ellipsoid is 0.4 mm. The boundary condition for the alternative pressure at the inlet is a fixed value of 5.5, while at the outlet, it is a fixed gradient of 0. The boundary conditions for other parameters at the inlet and outlet are a fixed gradient of 0, zero vector, or zero tensor.

**TABLE 3:** Shape parameters for the embolization test in the stenotic pipe.

Shape	Parameters	Surface area^a^	Values
Torus	r,R	$4\pi^{2}\mathrm{rR}$	r=0.1,R=0.3
Disk	$h_{D},r_{D}$	$2\pi r_{D}\left(r_{D}+h_{D}\right)$	$h_{D}=0.1772,r_{D}=0.3545$
Sphere	$r_{S}$	$4\pi r_{S}^{2}$	$r_{S}=0.3070$
Ellipsoid	$a_{E},b_{E},c_{E}$	$4\pi {\left(\frac{a_{E}^{p}a_{E}^{p}+a_{E}^{p}c_{E}^{p}+b_{E}^{p}c_{E}^{p}}{3}\right)}^{\frac{1}{p}}$	$p=1.6075,b_{E}=0.4$ $a_{E}=c_{E}=0.2632$

^a^ The surface area of the ellipsoid was calculated with the approximated equation.

**FIGURE 11:**
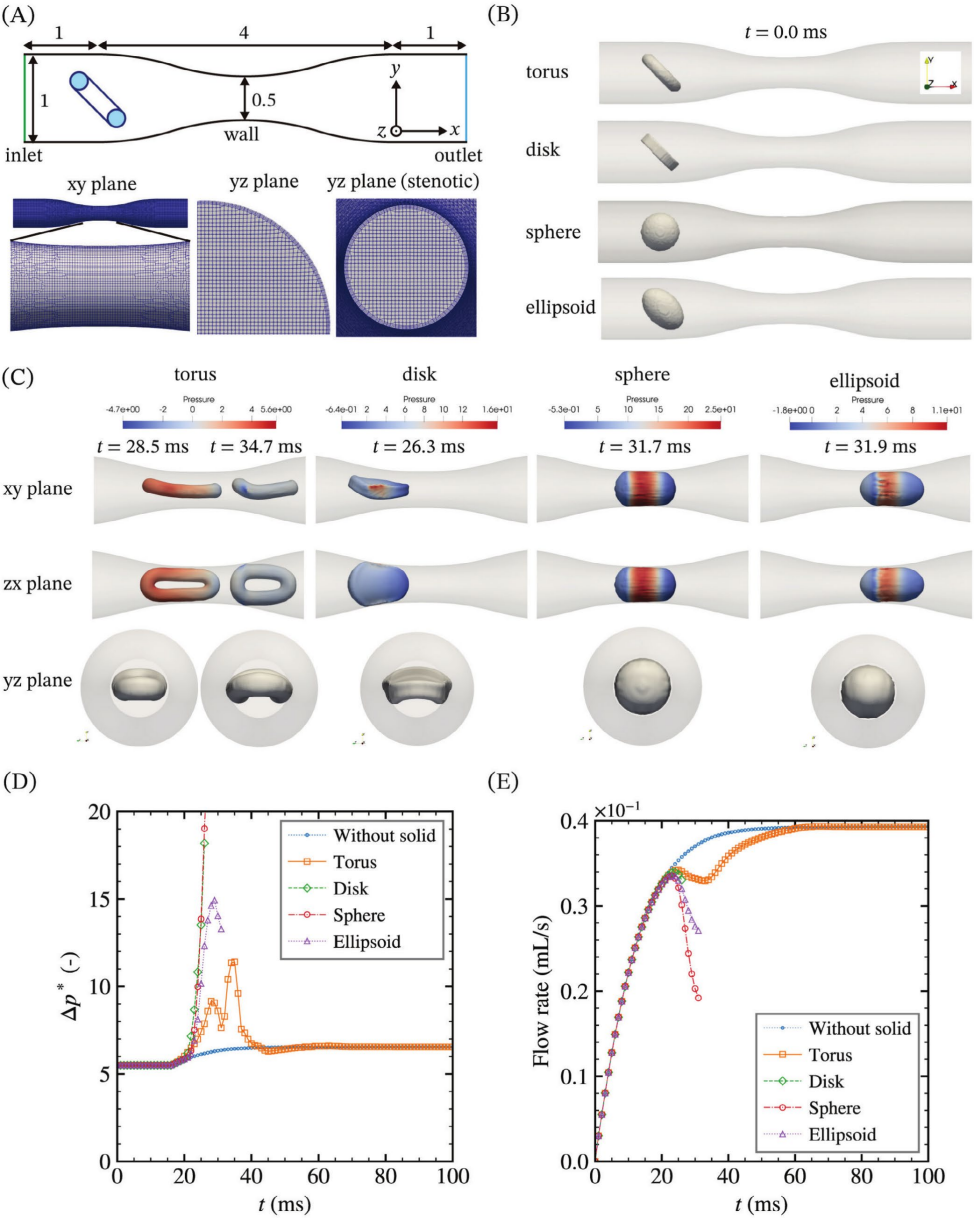
Evaluation of embolization with various shapes of microparticles in a stenotic pipe. (A) Schematic illustration of the stenotic pipe flow and the mesh structure of the geometry. (B) Initial geometry and solid shape of the microparticles at t=0 ms. All microparticles have the same surface areas. (C) Shape comparison of four kinds of microparticles at peak pressure. (D) Maximum pressure drop across the system. (E) Flow rate at the outlet of the stenotic pipe. Data depletion indicates computational divergence due to a rapid pressure increase near the wall. Torus shows the lowest pressure drop and smallest decrease in flow rate at outlet, while the other shapes of microparticles are diverged due to the rapid pressure increase.

Figure 11 shows the flow behavior of each microparticle within the stenotic pipe for the evaluation of embolization. All microparticles are inclined at 45° and centered at $y_{0}=0.5$. Figure 11C depicts the isosurface of each microparticle at the moment when the pressure drop reaches the local maximum as shown in Figure 11D. The torus deforms as it passes through the stenotic pipe and exhibits two local maxima of pressure drop during passage. In contrast, the other microparticle shapes cause computational divergence due to the rapid pressure increase near the wall as shown in Figure 11D. The reduction in flow rate at the outlet indicates the degree of embolization, and the torus shows the smallest decrease in flow rate among the microparticle shapes. When the torus is compressed from the radial direction, yield stresses concentrate in the compressed and deflated areas in the meridian direction, resulting in bending deformation under relatively low forces (Figure S6). In contrast, microparticles of the other shapes do not exhibit stress concentration and require larger force to deform, resulting in high pressure near the contact area, which causes computational divergence. Specifically, the disk undergoes compression buckling upon entering the stenotic region (Figure S7). Furthermore, the ellipsoidal microparticle exhibits a relatively lower pressure drop and flow rate reduction compared to the spherical microparticle.

This result suggests that deformable ellipsoidal particles can reach distal blood vessels, consistent with the findings of a previous experimental study using capsule‐like microparticles [10]. In that study, Luo et al. compared the embolization endpoints of spherical and capsule‐shaped microparticles in a decellularized rabbit liver model. The capsule‐like microparticles were able to embolize more distally due to their lower viscous resistance. Furthermore, their broader contact area with the vessel wall enabled effective embolization with fewer particles. Similarly, toroidal microparticles exhibit an increased contact area when compressed by the vessel wall, which may facilitate distal embolization and reduce the risk of off‐target embolization. These shape‐dependent interactions highlight the potential advantages of nonspherical embolic agents in achieving targeted and efficient embolization. Therefore, these results indicate that the torus shows the lowest pressure drop and flow rate reduction due to deformation among these shapes with the same surface area.

In this study, we investigated the flow and deformation behavior of the torus and evaluated the feasibility of novel embolic agents in blood vessels. Conventional TAE and its derivatives present complications due to embolization, and new embolic agents are expected in clinical use. We hypothesized that deformable toroidal microparticles are effective for satisfying the requirements and performed numerical calculations using a full Eulerian FSI implemented in OpenFOAM. The FSI solver based on *interIsoFoam* demonstrates sufficient accuracy in cases of solid deformation in cavity flow, and the errors of the solid centroid were inversely proportional to the number of grid points. While various shapes of microparticles can be fabricated using several techniques, their application and numerical studies for TAE are limited, and many studies focus on the flow of undeformed Lagrangian particles [6]. The flow and anisotropic deformation analysis using Full Eulerian FSI would be a valuable tool for addressing the viscoelastic properties of new embolic agents. A torus in a cylindrical pipe underwent various deformation modes such as rotation, bending, and elongation in laminar flow at low Reynolds numbers and converged to different steady states depending on initial orientation and position. Notably, the angle of inclined tori reached approximately 80°, and the velocity of inclined tori exceeds that of a horizontal torus centered along the pipe centerline. Deformation analysis of the cross‐section revealed that anisotropic deformation and bending angle were influenced by initial conditions, size, aspect ratio, and viscoelastic properties. Furthermore, the equilibrium position of the torus was determined by the balance of lift forces and became complex due to changes in the rotational radius over time. Vortex visualization and pressure gradient elucidated the mechanism by which deformation increased the distance to the wall, thereby reducing energy loss through vortex generation. Finally, toroidal microparticles showed a smaller pressure drop and flow rate reduction as they passed through the stenotic pipe compared to other microparticle shapes. Toroidal microparticles would be useful for controlling blood pressure and blood flow more sophisticatedly than conventional embolic reagents. Their shape‐induced deformation dynamics may improve anchoring within distal blood vessels and reduce the pressure drop that arises from embolization. Additionally, the inside of the torus can serve as a reservoir for therapeutic agents, including drugs, nanoparticles, or radioactive compounds. This feature enables multifunctional use in chemoembolization (TACE), radioembolization (TARE), and diagnostic imaging. Moreover, the ability to modulate local hemodynamics may help prevent complications such as hypoxia and local hypertension, contributing to safer and more effective embolization therapy. Our previous work demonstrated the fabrication of toroidal alginate microparticles with a characteristic size of approximately 200 μm [12]. Alginate microparticles have been proposed as potential embolic agents in recent studies [43, 44, 45, 46]. Accordingly, future experimental validation of the present numerical findings could be pursued through in vitro and in vivo investigations using similarly shaped particles. Such validation would provide critical insights into the physical behavior of viscoelastic embolic agents and support the clinical translation of shape‐ and material‐dependent embolization strategies.

## 4 Conclusions

Full Eulerian FSI has been implemented in OpenFOAM to numerically evaluate deformable toroidal embolic agents for localized medicine in blood vessels. Our flow and deformation analysis indicates that particle deformation affects their equilibrium states of both fluid and solid. In addition, vortex visualization demonstrated that deformation broadened the distance between the particle interface and pipe wall, resulting in the lower pressure drop. The embolization model with a stenotic pipe revealed that the torus suppressed the pressure increase due to the gap between the wall and the buckling process with the concentration of yield stress compared with the other three particles with the same surface area. Commercially available embolic agents have various ranges of viscoelasticity and show different therapeutic effects in clinical studies. Therefore, our computational method is expected to be a useful approach to evaluate the flow and deformation behavior of not only toroidal embolic agents but also conventional and nonspherical embolic agents in the future.

## Nomenclature

a,b,c
: Harf length of ellipsoid
B
: Left Cauchy‐Green deformation tensor
$\tilde{B}$
: Corrected left Cauchy‐Green deformation tensor
Co
: Courant number
D
: Cylinder diameter
D
: Shear rate tensor
g
: Gravity acceleration vector
G
: Shear modulus
$G\left(t\right)$
: Gyration tensor
h
: Height
I
: Unit tensor
K
: Eigenfrequency parameter
L
: Cylinder length
$\left\|L_{2}\right\|$
: $L_{2}$ norm
$\left\|L_{\infty }\right\|$
: $L_{\infty }$ norm
n
: Orientation vector
N
: Number of cells
p
: Pressure
$p_{\mathrm{rgh}}$
: Alternative pressure
Δp
: Pressure drop
Q
: Second invariant of velocity gradient tensor (Q‐criterion)
r
: Minor radius
R
: Major radius
Re
: Reynolds number
S
: Deformation rate tensor
S
: Source term
t
: Time
$u={\left[u_{x},u_{y},u_{z}\right]}^{T}$
: Velocity vector
V
: Volume
w
: Width
$x={\left[x,y,z\right]}^{T}$
: Position vector in current coordinates
X,Y
: Two‐dimensional coordinate system

### Superscript

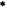

Dimensionless property

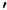

Deviatoric tensor

### Subscript

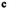

Centroid

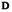

Disk

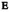

Ellipsoid

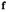

Fluid

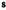

Solid

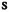

Sphere

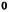

Initial state

### Greek Letters

α
: Volume fraction of solid
ζ
: Local coordination axis
η
: Local coordination axis
θ
: Angle of the ellipse in two‐dimensional coordinate system
μ
: Viscosity
κ
: Circular eigenfrequency
ν
: Kinematic viscosity
ξ
: Local coordination axis
ρ
: Density
σ
: Cauchy stress tensor
ψ
: Angle between orientation vector and unit vector of x‐axis
Ψ
: Reconstruct difference function
Ω
: Vorticity tensor

## Author Contributions

**Kazuki Matsumiya:** conceptualization, data curation, formal analysis, funding acquisition, investigation, methodology, resources, software, visualization, writing – original draft. **Natsuko F. Inagaki:** writing – review and editing. **Taichi Ito:** conceptualization, resources, supervision, writing – review and editing. **Kazuyasu Sugiyama:** methodology, supervision, writing – review and editing. **Shu Takagi:** methodology, supervision, writing – review and editing.

## Ethics Statement

The authors have nothing to report.

## Conflicts of Interest

The authors declare no conflicts of interest.

## Supporting information


**Data S1:** Supporting Information.

**Data S2:** Supporting Information.

**Data S3:** Supporting Information.

**Data S4:** Supporting Information.

## Data Availability

The data that support the findings of this study are available from the corresponding author upon reasonable request.
