# Supplementary material for: Computational Fluid Dynamics of the Flow of the Deformable Toroidal Embolic Agents Within Straight and Stenotic Pipes by Full Eulerian FSI Method
Source: Int J Numer Method Biomed Eng. 2025 Sep 8;41(9):e70089. doi: 10.1002/cnm.70089 (PMC12416352; doi:10.1002/cnm.70089)
Supplement: Supplementary file 1 — Data S1: Supporting Information. [file CNM-41-e70089-s004.docx]

Supporting Information

Computational fluid dynamics of the flow of the deformable toroidal embolic agents within straight and stenotic pipes by full Eulerian FSI method

*Kazuki Matsumiya^1^, Kazuyasu Sugiyama^2*^, Natsuko F. Inagaki^1^,*

*Shu Takagi^3,4*^, Taichi Ito^1,3,5^*

^1^ Department of Chemical System Engineering, School of Engineering, The University of Tokyo, Tokyo, Japan.

^2^ Department of Mechanical Science and Bioengineering, Graduate School of Engineering Science, The University of Osaka, Osaka, Japan.

^3^ Department of Bioengineering, School of Engineering, The University of Tokyo, Tokyo, Japan.

^4^ Department of Mechanical Engineering, School of Engineering, The University of Tokyo, Tokyo, Japan.

^5^ Department of Radiology and Biomedical Engineering, School of Medicine, The University of Tokyo, Tokyo, Japan.

***Corresponding Author**

Double corresponding Authors

* Shu Takagi (E-mail: takagi@mech.t.u-tokyo.ac.jp)

* Kazuyasu Sugiyama (E-mail: sugiyama.kazuyasu.es@osaka-u.ac.jp)

**Contents**

**Figure S1.** Deformation analysis of the cross-section of the inclined tori.

**Figure S2.** The effects of the centroid velocity on the vibration frequency of a torus.

**Figure S3.** Deformation analysis of bending and elongation of the inclined tori.

**Figure S4.** The effects of the viscosity of a torus.

**Figure S5.** The effects of the size of a torus.

**Figure S6.** Pressure distribution on the cross-section of the deformed torus in a stenotic region.

**Figure S7.** Deformation of the disk-shaped microparticles in the stenotic region.


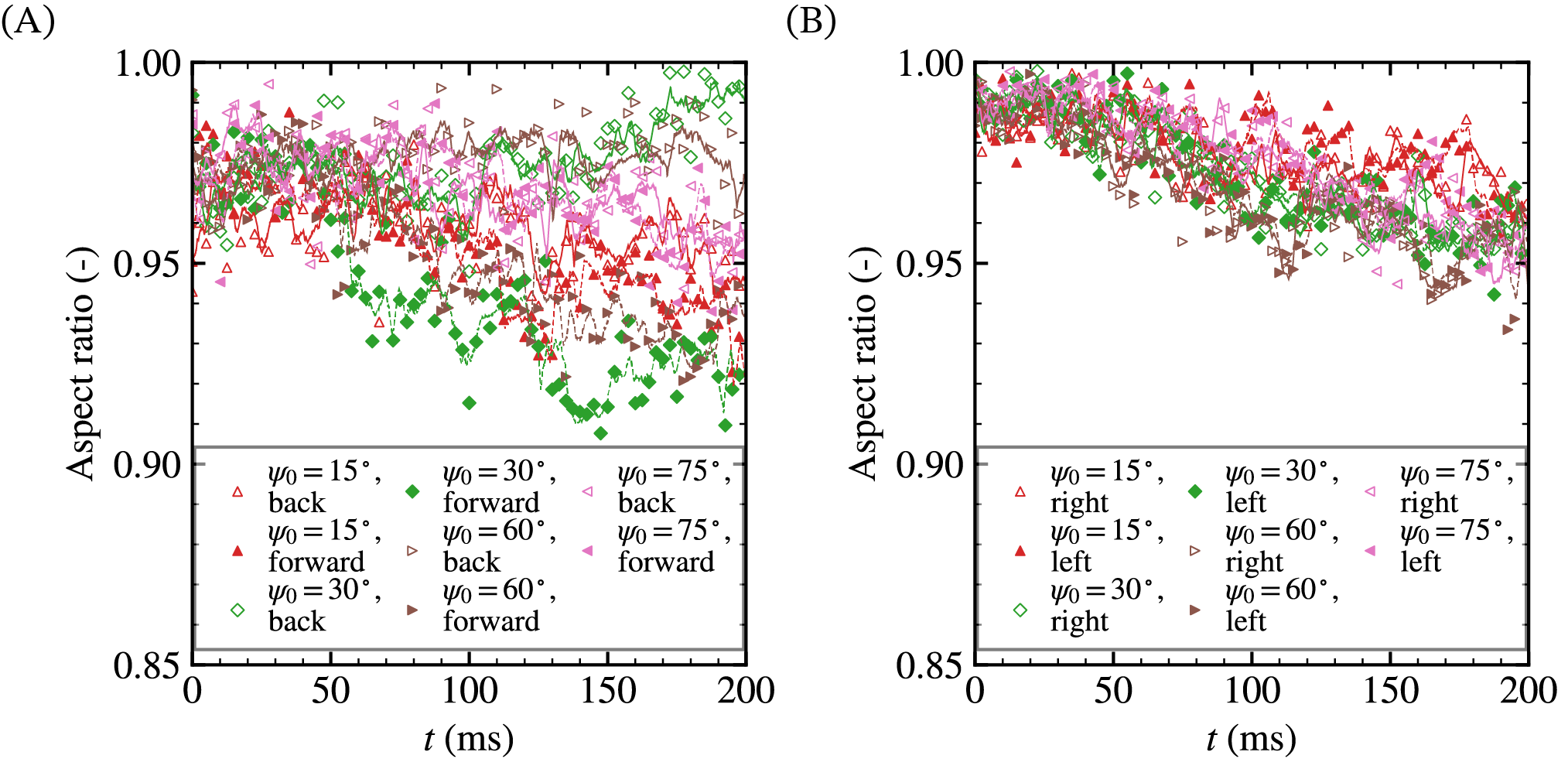


Figure S1. Deformation analysis of the cross-section of the inclined tori ($\boldsymbol{\psi}_{\boldsymbol{0}}\boldsymbol{=1}\boldsymbol{5}^{\boldsymbol{\circ}}\boldsymbol{,3}\boldsymbol{0}^{\boldsymbol{\circ}}\boldsymbol{, 6}\boldsymbol{0}^{\boldsymbol{\circ}}\boldsymbol{,7}\boldsymbol{5}^{\boldsymbol{\circ}}$). Time course changes of the aspect ratios of the isolines on each cross-section. The aspect ratios of the isolines are obtained by elliptic approximation. The solid and dashed lines represent the moving average of up to twenty-five data points ($\Delta t=2.5$ ms).


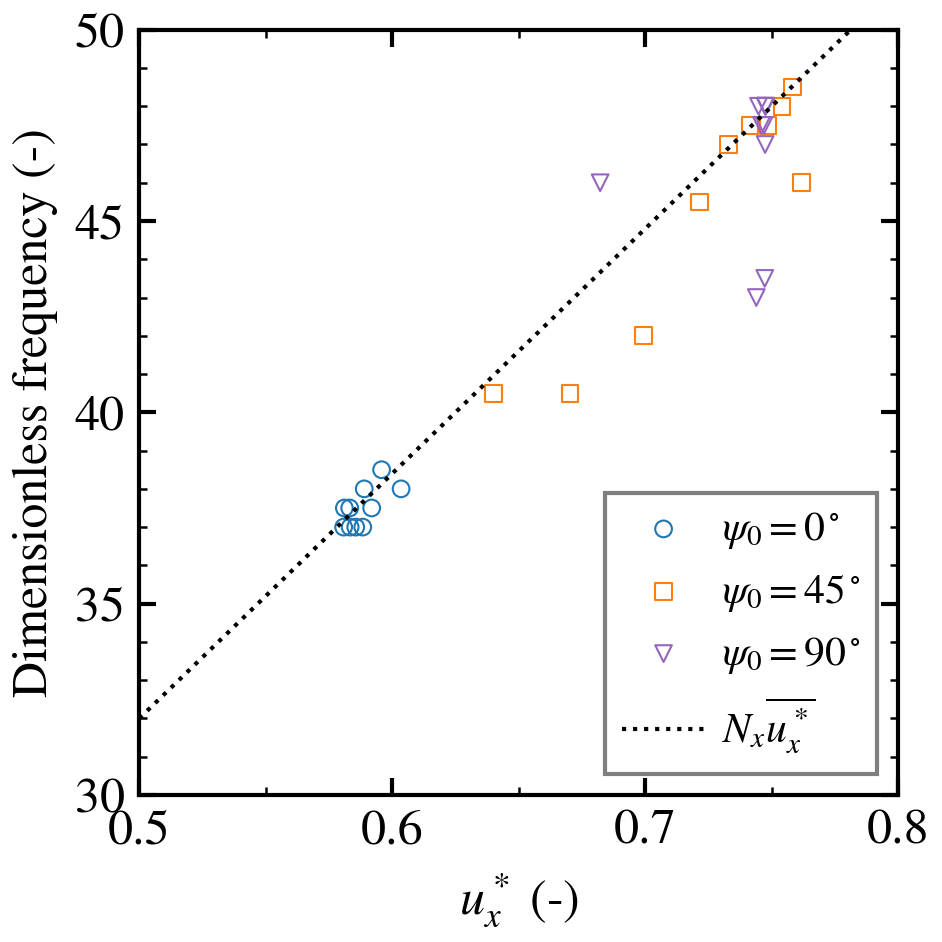


Figure S2. The effects of the centroid velocity on the vibration frequency of the torus. Dimensionless frequencies were derived via Fourier transformation of the cross-sectional vibration data of the vertical torus in the meridian direction. The time interval between data points was 0.1 ms, with 200 data points per interval used for the Fourier transformation.


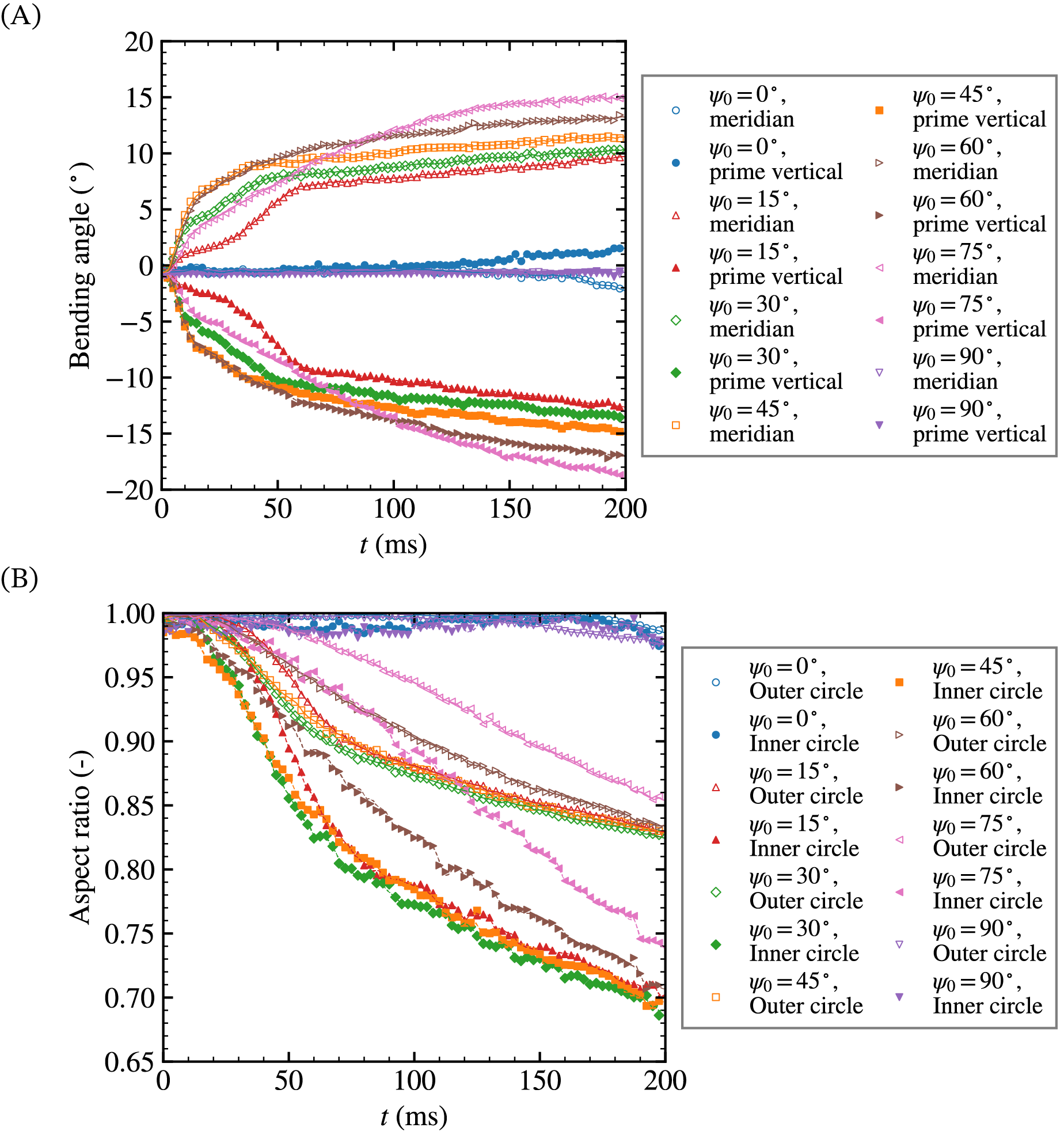


Figure S3. Deformation analysis of bending and elongation of the inclined tori. Time course changes of (A) the bending angles and (B) the aspect ratios of the projected circles of the inclined tori. The solid and dashed lines represent the moving average of up to twenty-five data points ($\Delta t=2.5$ ms).


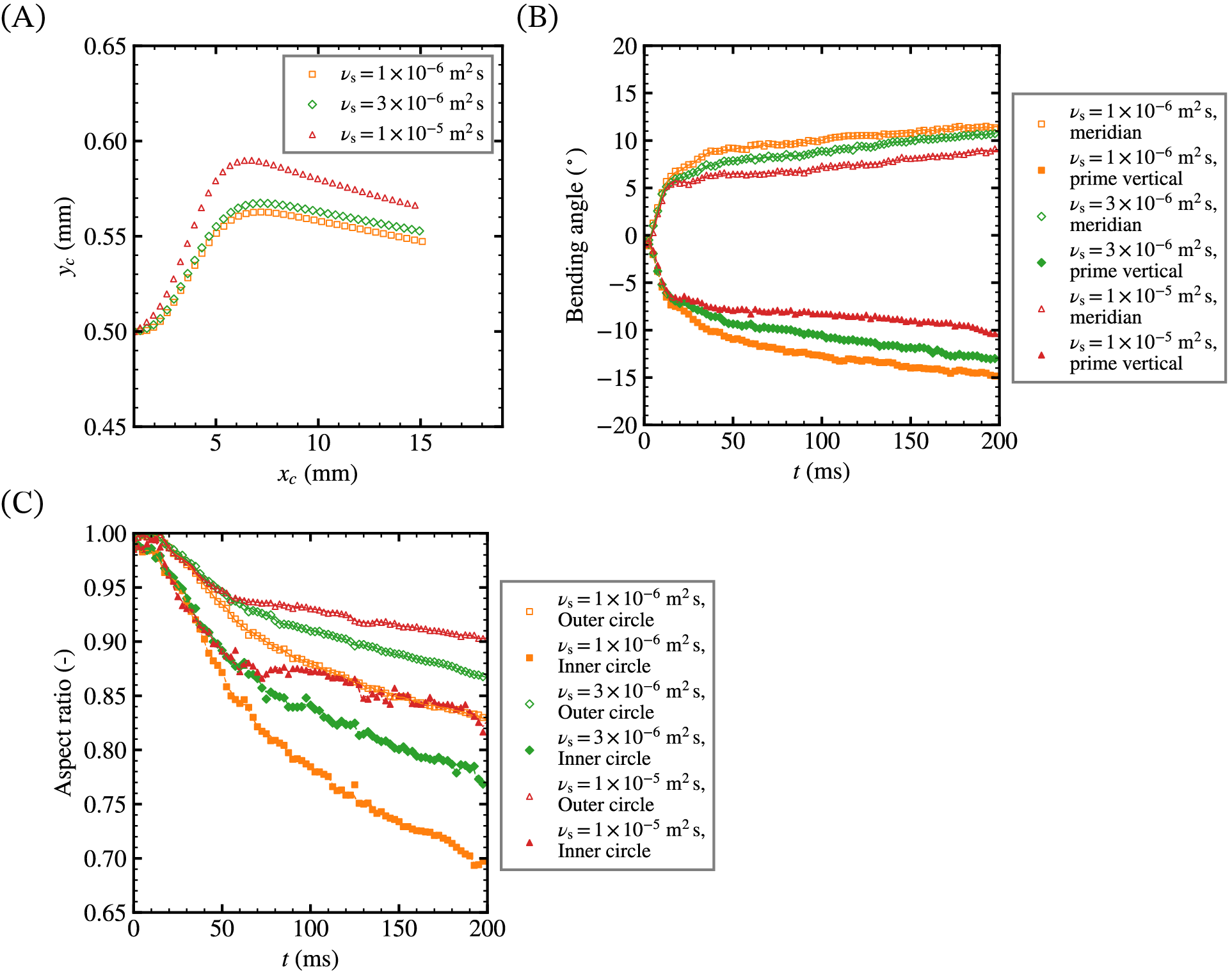


Figure S4. The effects of the viscosity of a torus. Time course changes of (A) the position of the centroid, (B) the bending angle on each cross-section, and (C) the aspect ratios of the inner and outer circles of the projected rings. The solid and dashed lines represent the moving average of up to twenty-five data points ($\Delta t = 2.5$ ms).


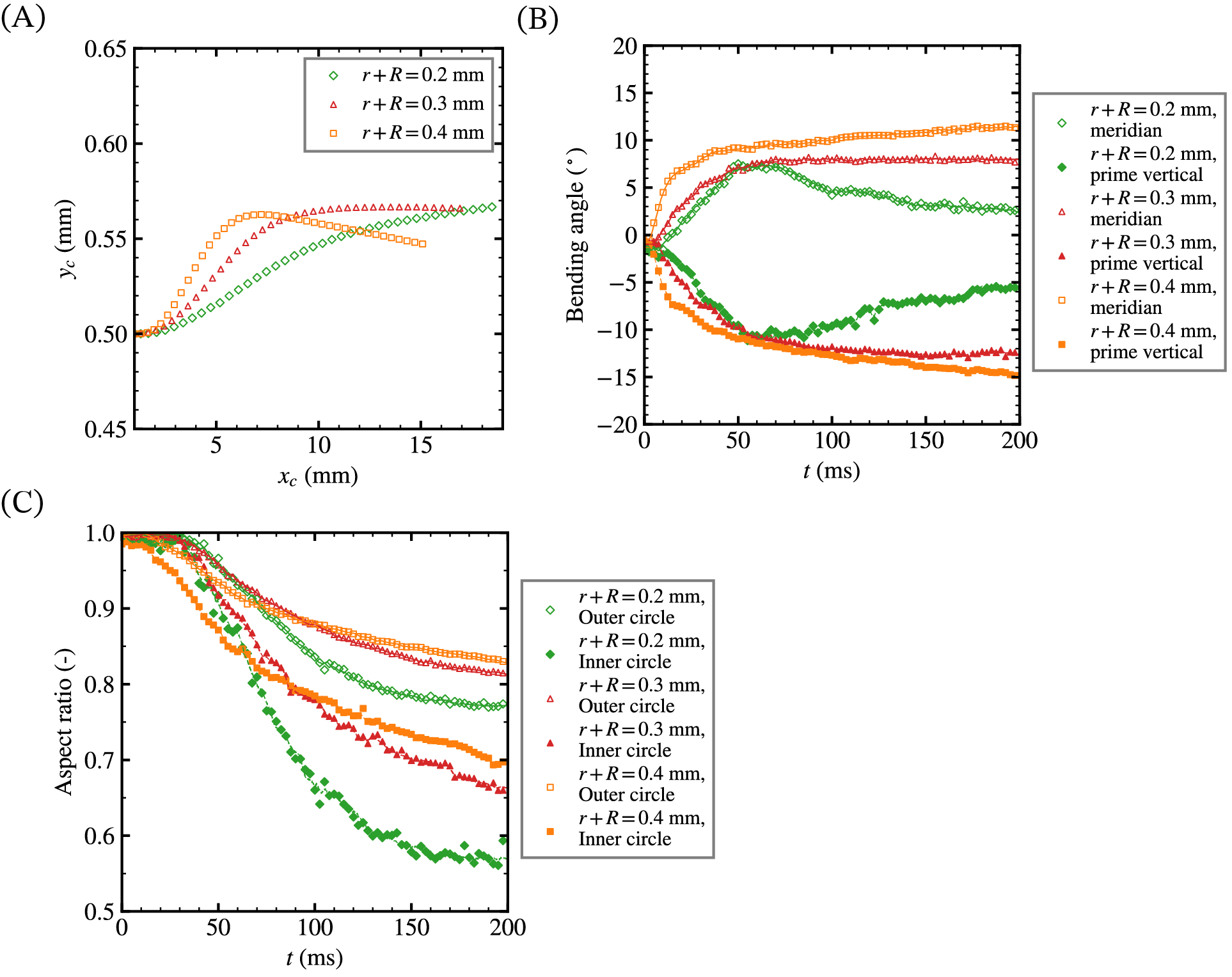


Figure S5. The effects of the size of a torus. Time course changes of (A) the position of the centroid, (B) the bending angle on each cross-section, and (C) the aspect ratios of the inner and outer circles of the projected rings. The solid and dashed lines represent the moving average of up to twenty-five data points ($\Delta t = 2.5$ ms).


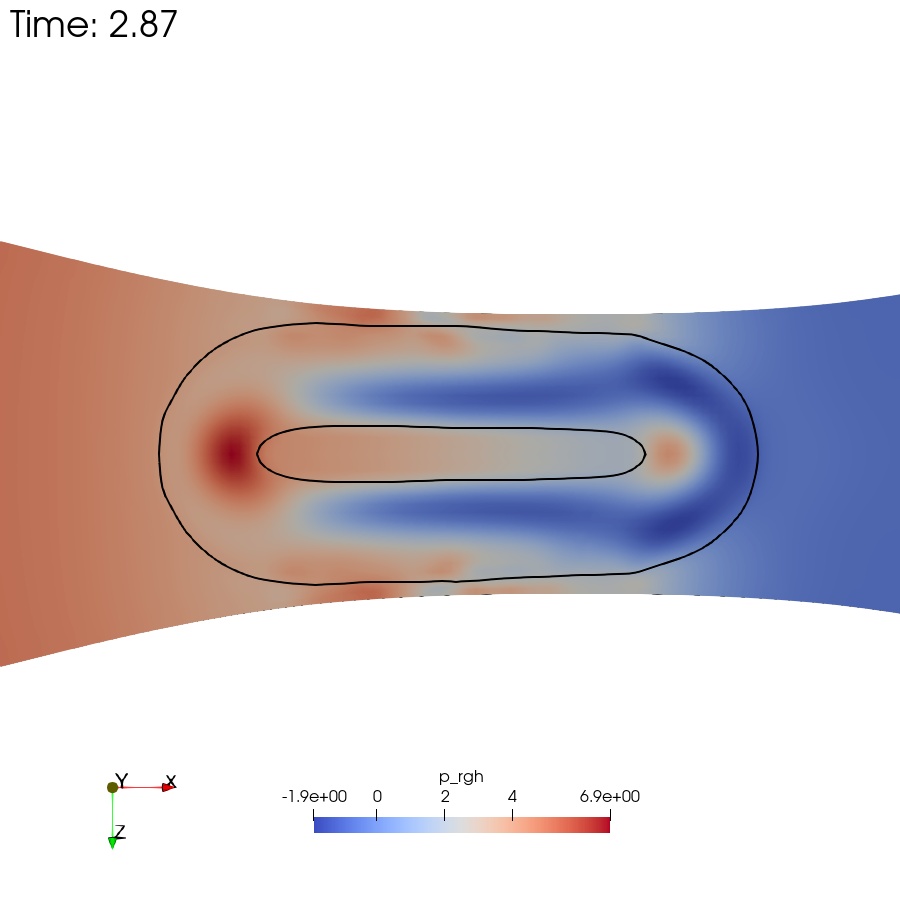


Figure S6. Pressure distribution on the cross-section of the deformed torus in a stenotic region. Color bar depicts the alternative pressure in the system. The black solid line represents the isoline of volume fraction of the deformed torus at $\alpha=0.5$.


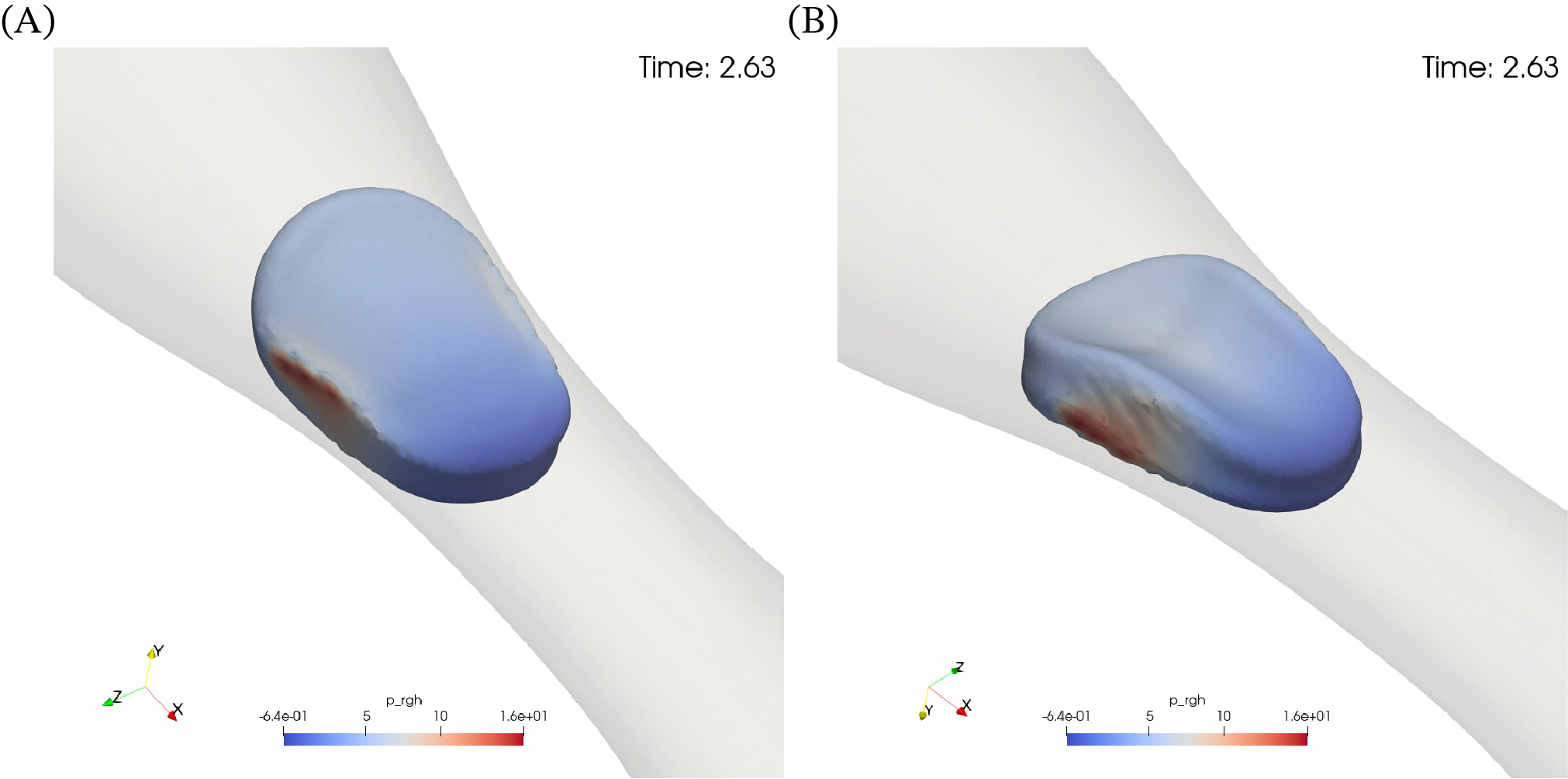


Figure S7. Deformation of the disk-shaped microparticles in the stenotic region. (A) Front and (B) Back side views of the isosurface of volume fraction of the disk at $\boldsymbol{\alpha=0.5}$. The color bar depicts the alternative pressure on the isosurface.

Movie S1. Temporal change of shape and orientation of the inclined torus ($\psi_0$ = 45 degree) in Hagen-Poiseuille flow viewed from the yz-plane (top) and xy-plane (bottom).

Movie S2. Temporal change of shape and orientation of the horizontal torus ($\psi_0$ = 90 degree) in Hagen-Poiseuille flow viewed from the yz-plane (top) and xy-plane (bottom).

Movie S3. Temporal change of shape and orientation of the vertical torus ($\psi_0$ = 0 degree) in Hagen-Poiseuille flow viewed from the yz-plane (top) and xy-plane (bottom).
